# Asymmetric α-amination of β-keto esters using a guanidine–bisurea bifunctional organocatalyst

**DOI:** 10.3762/bjoc.12.22

**Published:** 2016-02-04

**Authors:** Minami Odagi, Yoshiharu Yamamoto, Kazuo Nagasawa

**Affiliations:** 1 Department of Biotechnology and Life Science, Tokyo University of Agriculture and Technology, 2-24-16, Naka-cho, Koganei city, 184-8588, Tokyo, Japan

**Keywords:** α-amination, bifunctional catalyst, guanidine, hydrogen-bonding catalyst, urea

## Abstract

An asymmetric α-amination of β-keto esters with azodicarboxylate in the presence of a guanidine–bisurea bifunctional organocatalyst was investigated. The α-amination products were obtained in up to 99% yield with up to 94% ee.

## Introduction

Asymmetric α-amination of β-keto esters is an important synthetic route to optically active α-amino acid derivatives with chiral quaternary stereocenters [1–2]. Since an α-amino acid moiety is frequently found in biologically active compounds, considerable efforts have been made to achieve a stereoselective synthesis of this structure [3–4]. In particular, catalytic asymmetric α-amination of β-keto esters has been widely explored, using both metal catalysts and organocatalysts [5–18].

**Figure 1:**
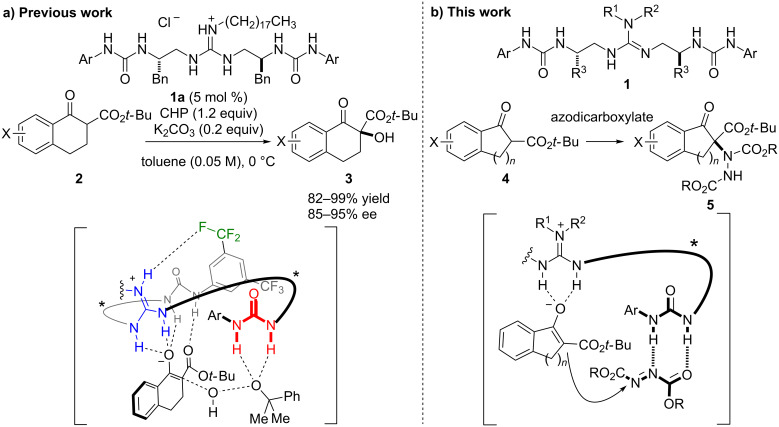
a) Asymmetric α-hydroxylation of **2** in the presence of **1a**. b) Asymmetric α-amination of **4** explored in this study.

We have developed a series of guanidine–bis(thio)urea bifunctional organocatalysts, and have used them in a variety of asymmetric reactions [19–20]. Recently, we disclosed an α-hydroxylation of tetralone-derived β-keto esters **2** using guanidine–bisurea bifunctional organocatalyst **1a** in the presence of cumene hydroperoxide (CHP) as an oxidant (Figure 1a) [21]. This reaction provides the corresponding α-hydroxylation products **3** in high yield with high enantioselectivity. A computational study of the transition state of this reaction revealed that inter- and intramolecular hydrogen-bonding networks between catalyst and substrate are critical for obtaining high enantioselectivity [22]. Based upon these insights, we expected that guanidine–bisurea bifunctional organocatalyst **1** would be effective in promoting α-amination of β-keto esters as a result of interactions between guanidine and enolate of the β-keto ester, and between urea and azodicarboxylate (Figure 1b). Herein, we describe the catalytic asymmetric α-amination of β-keto esters with azodicarboxylates as a nitrogen source in the presence of **1**.

## Results and Discussion

The reaction conditions for α-amination of β-keto ester **4a** in the presence of diethyl azodicarboxylate (DEAD) were optimized as follows. First, we focused on the catalyst structure (Table 1) [23]. Initially, the R^3^ substituent on the chiral spacer of the catalyst **1** was optimized (Table 1, entries 1–4). The catalyst with a benzyl group at R^3^ (**1a**) afforded **5a** in excellent yield with moderate enantioselectivity for *R* configuration (Table 1, entry 1) [24–25]. When R^3^ was changed to a phenyl group, the enantioselectivity was slightly increased to 59% ee (Table 1, entry 2). In the case of a methyl group, **5a** was obtained in 98% yield with 50% ee (Table 1, entry 3). An isopropyl group as R^3^ group was most effective, affording **5a** with 66% ee (Table 1, entry 4). Next, we optimized R^1^ and R^2^ on the guanidine moiety (Table 1, entries 5 and 6). A catalyst bearing a six-membered ring at R^1^ and R^2^ (**1e**) gave excellent yield, but with only 27% ee (Table 1, entry 5). Interestingly, catalyst **1f** bearing a pyrrolidine ring at R^1^ and R^2^ showed the highest selectivity, and **5a** was obtained in 99% yield with 80% ee (Table 1, entry 6). Thus, we chose **1f** as the optimized catalyst for the reaction [26].

**Table 1:** Optimization of catalyst structure.^a^

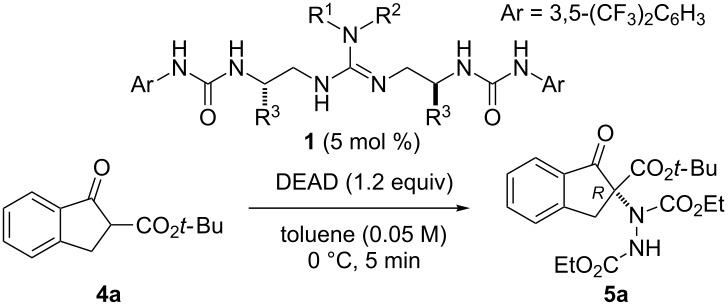						

entry	catalyst **1**				α-amination product **5a**	
		
		R^1^, R^2^	R^3^		yield (%)^b^	ee (%)^c^

1	**1a**	H, –(CH_2_)_17_CH_3_	Bn		99	53
2	**1b**	H, –(CH_2_)_17_CH_3_	Ph		94	59
3	**1c**	H, –(CH_2_)_17_CH_3_	Me		98	50
4	**1d**	H, –(CH_2_)_17_CH_3_	iPr		97	66
5	**1e**	–(CH_2_)_5_–	iPr		93	27
6	**1f**	–(CH_2_)_4_–	iPr		99	80

^a^Reaction conditions: **4a** (0.1 mmol), DEAD (0.12 mmol) and **1** (5 mol %) in toluene (2.0 mL) at 0 °C. ^b^Isolated yield. ^c^Determined by HPLC analysis using a chiral stationary phase. DEAD = diethyl azodicarboxylate.

Next, we investigated various solvents, such as ethyl acetate, dichloromethane, acetonitrile and diethyl ether (Table 2, entries 1–5) for the reaction in the presence of catalyst **1f** (Table 2). The best result was obtained with diethyl ether, and **5a** was isolated in 95% yield with 85% ee (Table 2, entry 5). The enantioselectivity was improved to 90% ee by decreasing the reaction temperature to −40 °C without decrease in the yield (Table 2, entry 6). When the reaction was performed at −78 °C, the yield of **5a** was dropped to 91% (Table 2, entry 7).

**Table 2:** Investigation of solvent effect.^a^

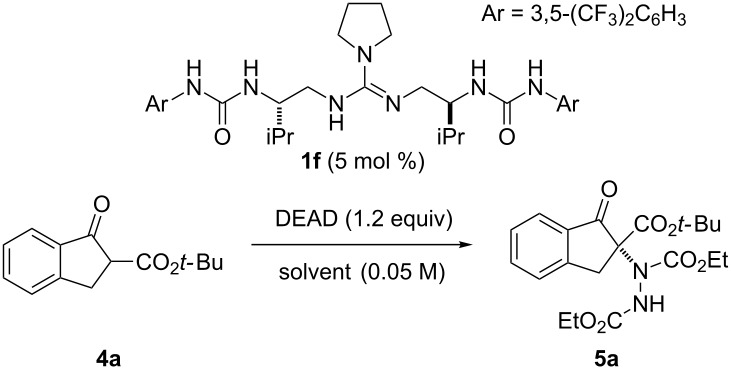					

entry	solvent	time (min)	temp (°C)	α-amination product **5a**	

				yield (%)^b^	ee (%)^c^

1	toluene	5	0	99	80
2	EtOAc	5	0	99	78
3	DCM	30	0	99	75
4	MeCN	30	0	97	58
5	Et_2_O	5	0	95	85
6	Et_2_O	5	−40	99	90
7	Et_2_O	30	−78	91	89

^a^Reaction conditions: **4a** (0.1 mmol), DEAD (0.12 mmol) and **1f** (5 mol %) in solvent (2.0 mL). ^b^Isolated yield. ^c^Determined by HPLC analysis using a chiral stationary phase. DEAD = diethyl azodicarboxylate. EtOAc = ethyl acetate. DCM = dichloromethane. MeCN = acetonitrile. Et_2_O = diethyl ether.

As a further investigation, we optimized the ester moiety of the azodicarboxylate (Table 3). In addition to the ethyl ester (Table 3, entry 1), we examined benzyl, isopropyl, and *tert*-butyl ester as azodicarboxylate (Table 3, entries 2–4). By changing the ethyl ester to a benzyl or isopropyl ester, the amination products **6a** and **7a** were obtained in excellent yield, but the enantioselectivity was dropped to 64 and 79% ee, respectively (Table 3, entries 2 and 3). In the case of the *tert*-butyl ester, the reactivity of the azodicarboxylate was drastically decreased, and the reaction has not been completed after 48 h. The enantioselectivity of **8a** was also poor (Table 3, entry 4).

**Table 3:** Optimization of the ester moiety of azodicarboxylate.^a^

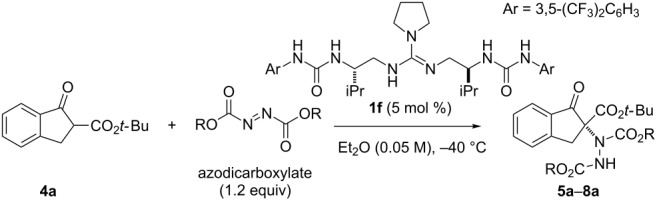					

entry	azodicarboxylate	time	α-amination product		
	
	R			yield (%)^b^	ee (%)^c^

1	Et	5 min	**5a**	99	90
2	Bn	5 min	**6a**	98	64
3	iPr	30 min	**7a**	98	79
4	*t-*Bu	48 h	**8a**	58	44

^a^Reaction conditions: **4a** (0.1 mmol), azodicarboxylate (0.12 mmol) and **1f** (5 mol %) in Et_2_O (2.0 mL) at −40 °C. ^b^Isolated yield. ^c^Determined by HPLC analysis using a chiral stationary phase.

**Scheme 1:**
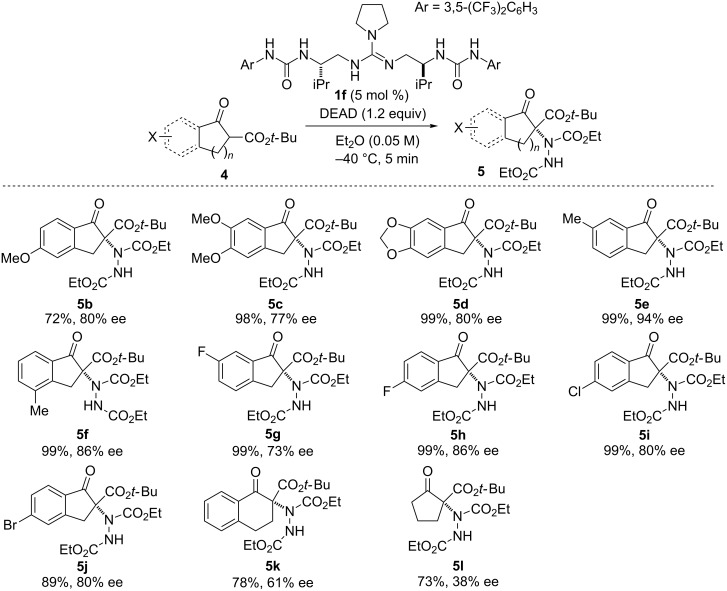
Substrate scope of α-amination.

With the optimal reaction conditions in hand (Table 2, entry 6), we investigated the substrate scope for α-amination of β-keto esters (Scheme 1). First, various indanone-derived β-keto esters were examined. With electron-donating substituents such as methoxy and methyl, the corresponding amination products **5b**–**f** were obtained in high yield (72–99%) with high enantioselectivity (77–94% ee). In the case of substrates bearing electron-withdrawing groups, such as halogen atoms, the amination products **5g**–**j** were obtained with high enantioselectivity (73–86% ee). On the other hand, in the case of tetralone derivative **4k** and cyclopentanone derivative **4l**, the enantioselectivity of the products **5k** and **5l** was moderate to low (61% ee and 38% ee, respectively).

**Figure 2:**
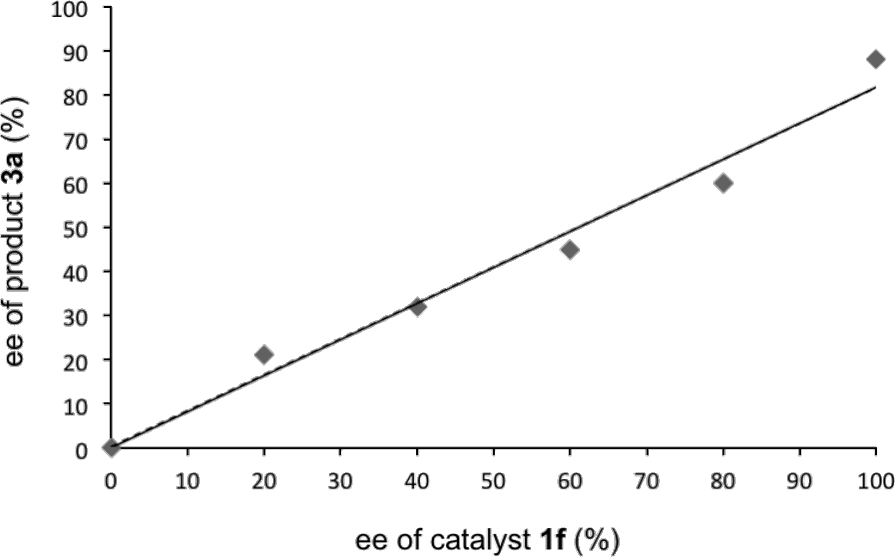
NLE study of α-amination.

**Scheme 2:**
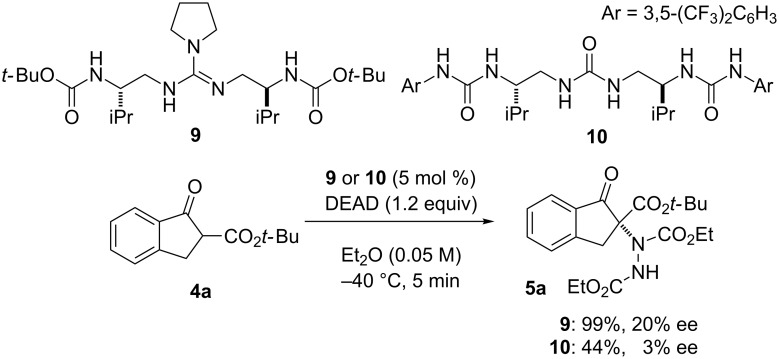
α-Amination of **4a** using **9** or **10** as catalyst.

To get insight into the transition state of the reaction, we performed a nonlinear effect (NLE) study (Figure 2) [27]. We found a linear relationship between % ee of **1f** and **5a** in the reaction. This result suggests that the stereoselectivity is controlled by the monomeric structure of **1f** [28–31]. Furthermore, to confirm the requirement of bifunctionality in catalyst **1**, we performed the α-amination reaction in the presence of carbamate **9** or triurea **10** as a catalyst (Scheme 2). In both cases, the enantioselectivity of the α-amination product **3a** was drastically decreased. These results clearly show that the guanidine and urea moieties in the catalyst **1f** are mandatory for obtaining high enantioselectivity, presumably interacting with the enolate of **4a** and DEAD, respectively.

## Conclusion

In conclusion, we have developed an asymmetric α-amination of β-keto esters **4** by using guanidine–bisurea bifunctional organocatalyst **1f** in the presence of diethyl azodicarboxylate (DEAD). The α-amination of various indanone-derived β-keto esters proceeded in high yield (up to 99% yield) and with high enantioselectivity (up to 94% ee).

## Supporting Information

File 1 Experimental procedures, copies of NMR spectra and HPLC chromatograms.
