# Supplementary material for: Asymmetric α-amination of β-keto esters using a guanidine–bisurea bifunctional organocatalyst
Source: Beilstein J Org Chem. 2016 Feb 4;12:198–203. doi: 10.3762/bjoc.12.22 (PMC4778535; doi:10.3762/bjoc.12.22)
Supplement: File 1 — Experimental procedures, copies of NMR spectra and HPLC chromatograms. [file Beilstein_J_Org_Chem-12-198-s001.pdf]

Supporting Information
for

Asymmetric α-amination of β-keto esters using a guanidine–bisurea bifunctional organocatalyst

Minami Odagi*[1], Yoshiharu Yamamoto[1] and Kazuo Nagasawa*[1]

Address: [1]Department of Biotechnology and Life Science, Tokyo University of Agriculture and Technology, 2-24-16, Naka-cho, Koganei city, 184-8588, Tokyo, Japan

Email: Minami Odagi - odagi@cc.tuat.ac.jp; Kazuo Nagasawa* - knaga@cc.tuat.ac.jp

*Corresponding author

Experimental procedures, copies of NMR spectra and HPLC chromatograms

Table of Contents:

1. Instrumentation S2

2. Details of optimization of reaction conditions for α-amination S3

3. General procedure for α-amination S5

4. Chiral HPLC analysis S9

5. ^{1}H and ^{13}C NMR spectra of new compounds S24

1. Instrumentation

Flash chromatography was performed using silica gel 60 (spherical, particle size 0.040–0.100 mm. Kanto Co., Inc., Tokyo, Japan). Optical rotations were measured on a JASCO P-2200 polarimeter (JASCO, Tokyo, Japan). ^{1}H and ^{13}C NMR spectra were recorded on JNM-AL300 and JNM-ECA500 instruments (JEOL, Tokyo, Japan). Chemical shifts in dimethylsulfoxide-d_6 were reported in the scale relative to residual dimethyl sulfoxide (2.50 ppm) for ^{1}H NMR. For ^{13}C NMR, chemical shift was reported in the scale relative to dimethylsulfoxide-d_6 (39.5 ppm) as an internal reference. Mass spectra were recorded on JEOL JMS-T100LC and JEOL JMA-HX110 spectrometer (JEOL, Tokyo, Japan). HPLC analysis on chiral stationary phase was performed on JASCO 800-series instruments (JASCO, Tokyo, Japan). Daicel Chiralpak AD-H and OD-H columns (Daicel, Osaka, Japan) with hexane/2-propanol and hexane/ethanol as the eluent were used.

2. Details of optimization of reaction conditions for α-amination

Table S1: Optimization for substituents of urea moiety.[a]

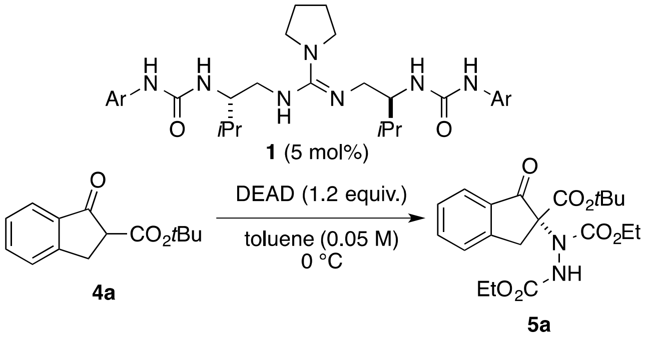

entry	catalyst 1		time (min)	α-amination product 5a	
		Ar		yield (%)[b]	ee (%)[c]
1	**1g**	3,5–$(CF_3)_2C_6H_3$	5	99	80
2	**1h**	4–$CF_3C_6H_4$	30	93	74
3	**1i**	3–$CF_3C_6H_4$	60	99	49
4	**1j**	2–$CF_3C_6H_4$	5	96	–24
5	**1k**	3,5–$(F)_2C_6H_3$	5	99	67
6	**1l**	3,5–$(MeO)_2C_6H_3$	60	91	42

[a]Reaction condition: **4a** (0.1 mmol), DEAD (0.12 mmol) and **1** (5 mol %) in toluene at 0 °C. [b]Isolated yield. [c]Determined by HPLC analysis using a chiral stationary phase.

Table S2: Optimization of catalyst structure for guanidine–bisthiourea bifunctional organocatalyst.[a]

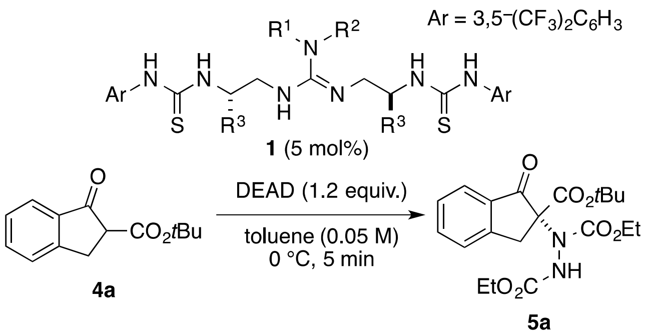

entry	catalyst 1			α-amination product 5a	
		R^1, R^2	R^3	yield (%)[b]	ee (%)[c]
1	**1m**	H, –$(CH_2)_{17}CH_3$	Bn	98	71

2	**1n**	H, –$(CH_2)_{17}CH_3$	Ph	96	64
3	**1o**	H, –$(CH_2)_{17}CH_3$	Me	96	65
4	**1p**	H, –$(CH_2)_{17}CH_3$	*i*Pr	99	60
5	**1q**	–$(CH_2)_5$–	Bn	99	14
6	**1r**	–$(CH_2)_4$–	Bn	99	–42

[a]Reaction condition: **4a** (0.1 mmol), DEAD (0.12 mmol) and **1** (5 mol%) in toluene at 0 °C. [b]Isolated yield. [c]Determined by HPLC analysis using a chiral stationary phase.

Table S3: Investigation of solvent effect.[a]

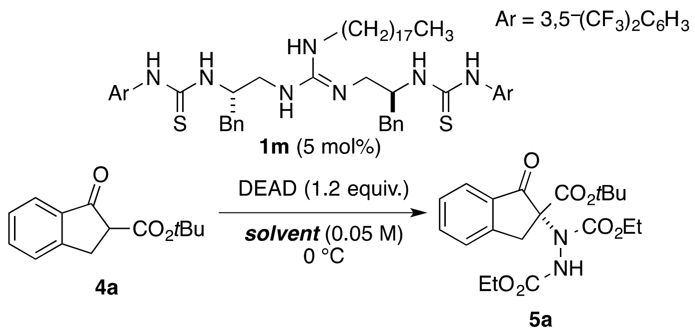

entry	solvent	time (min)	α-amination product **5a**	
			yield (%)[b]	ee (%)[c]
1	toluene	5	98	71
2	EtOAc	5	99	37
3	DCM	5	99	26
4	MeCN	30	99	7
5	Et_2O	5	99	64

[a]Reaction condition: **4a** (0.1 mmol), DEAD (0.12 mmol) and **1m** (5 mol%) in toluene at 0 °C. [b]Isolated yield. [c]Determined by HPLC analysis using a chiral stationary phase.

3. General procedure for α-amination

*Asymmetric α-amination of **4a** in the presence of **1f***

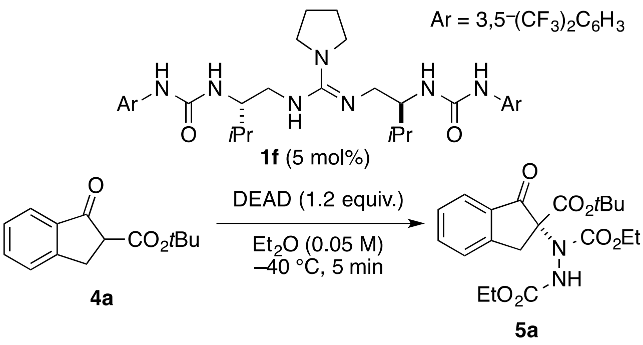

To a solution of β-keto ester **4a** (23.2 mg, 0.100 mmol) and guanidine–bisurea bifunctional organocatalyst **1f** (1.0 mg, 0.005 mmol) in diethyl ether (2 mL) was added dropwise diethyl azodicarboxylate (2.2 M in toluene, 55 μL, 0.120 mmol) at –40 °C. After being stirred for 5 min, the reaction mixture was quenched with saturated NH_4Cl solution. The organic phase was separated. Then, the aqueous phase was extracted with ethyl acetate three times. The combined organic solution was dried over $MgSO_4$, and concentrated in vacuo. The residue was purified by column chromatography on SiO_2 (ethyl acetate/hexane 1:10 to 1:1) to give α-amination product **5a** (40.5 mg, 99%) as a colorless amorphous. Enantiomeric excess was determined by HPLC analysis.

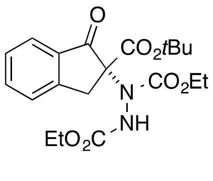

5a: $[\alpha]^{25}_{D}$ = −72.4 (*c* 3.95, $CHCl_3$, 90% ee); HPLC analysis: Daicel Chiralpak OD-H, hexane/2-propanol = 95:5, flow rate = 1.0 mL/min, τ_1 (minor) = 11.1, τ_2 (major) = 15.5.

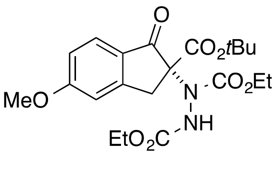

5b: $[\alpha]^{25}_{D}$ = −90.9 (*c* 3.60, $CHCl_3$, 80% ee); HPLC analysis: Daicel Chiralpak AD-H, hexane/2-propanol = 90:10, flow rate = 1.0 mL/min, τ_1 (minor) = 21.7, τ_2 (major) = 28.5.

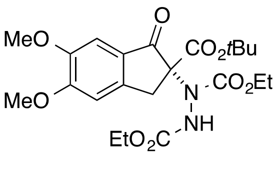

5c: $[\alpha]^{25}_{D}$ = −51.5 (*c* 3.98, $CHCl_3$, 77% ee); HPLC analysis: Daicel Chiralpak AD-H, hexane/2-propanol = 90:10, flow rate = 1.0 mL/min, τ_1 (minor) = 27.3, τ_2 (major) = 37.8.

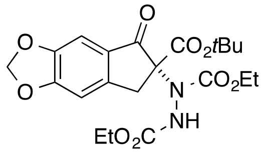

5d: $[\alpha]^{25}_{D}$ = −112.3 (*c* 3.45, $CHCl_3$, 80% ee); HPLC analysis: Daicel Chiralpak OD-H, hexane/2-propanol = 90:10, flow rate = 1.0 mL/min, τ_1 (minor) = 13.2, τ_2 (major) = 18.4; ^1H NMR (300 MHz, DMSO-*d6*, 100 °C) δ 7.03 (s, 1H), 7.01 (s, 1H), 6.13 (s, 2H), 4.17-3.91 (m, 4H), 3.86 (d, *J* = 17.2 Hz, 1H), 3.5 (d, *J* = 17.2 Hz, 1H), 1.36 (s, 9H), 1.18 (t, *J* = 6.9 Hz, 3H), 1.09 (br, 3H), 7.51 (d, *J* = 7.3 Hz, 1H); ^{13}C NMR (125 MHz, DMSO-*d6*, 100 °C) δ 191.02, 155.61, 154.62, 154.32, 147.81, 127.31, 104.88, 102.08, 101.6, 81.57, 78.54, 61.54, 60.36, 26.86, 13.58; HRMS (ESI, M+Na) calcd for 473.1536, found 473.1556.

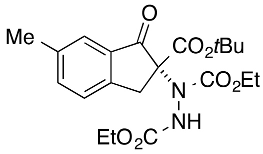

5e: $[\alpha]^{25}_{D}$ = −70.8 (*c* 4.74, $CHCl_3$, 94% ee); HPLC analysis: Daicel Chiralpak OD-H, hexane/2-propanol = 98:2, flow rate = 1.0 mL/min, τ_1 (minor) = 30.8, τ_2 (major) = 35.5; ^1H NMR (500 MHz, DMSO-*d6*, 100 °C) δ 7.5 (d, *J* = 8.0 Hz, 1H), 7.46 (s, 1H), 7.46 (s, 1H), 7.44 (d, *J* = 7.5 Hz, 1H), 4.15-3.86 (m, 5H), 3.57 (d, *J* = 16.0 Hz, 1H), 2.36 (s, 3H), 1.35 (s, 9H), 1.21-0.99 (m, 6H); ^{13}C NMR (125 MHz, DMSO-*d6*, 100 °C) δ 193.19, 155.60, 154.59, 136.82, 136.20, 133.26, 125.40, 123.39, 81.69, 78.56, 61.56, 60.34, 26.83, 19.84, 13.55; HRMS (ESI, M+Na) calcd for 443.1794, found 443.1759.

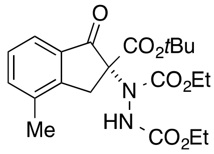

5f: $[\alpha]^{25}_{D}$ = −98.5 (*c* 4.16, $CHCl_3$, 86% ee); HPLC analysis: Daicel Chiralpak OD-H, hexane/2-propanol = 98:2, flow rate = 1.0 mL/min, τ_1 (minor) = 19.1, τ_2 (major) = 21.6; ^1H NMR (300 MHz, DMSO-*d6*, 100 °C) δ 7.51 (d, *J* = 7.3 Hz, 1H), 7.5 (d, *J* = 7.3 Hz, 1H), 7.33 (t, *J* = 7.3 Hz, 1H), 4.15-3.83 (m, 5H), 3.5 (d, *J* = 17.3 Hz, 1H), 2.34 (s, 9H), 1.18 (t, *J* = 6.9 Hz, 3H), 1.07 (br, 3H); ^{13}C NMR (75 MHz, DMSO-*d6*, 100 °C) δ 193.44, 155.59, 154.61, 135.34, 134.67, 132.89, 127.23, 121, 91.74, 78.54, 61.59, 60.34, 59.89, 26.81, 16.51, 13.55; HRMS (ESI, M+Na) calcd for 443.1794, found 443.1809.

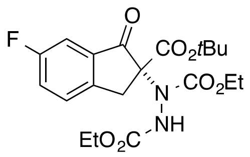

5g: $[\alpha]^{25}_{D}$ = −44.2 (*c* 3.29, $CHCl_3$, 73% ee); HPLC analysis: Daicel Chiralpak OD-H, hexane/2-propanol = 95:5, flow rate = 1.0 mL/min, τ_1 (minor) = 9.8, τ_2 (major) = 12.2; ^{1}H NMR (500 MHz, DMSO-*d6*, 100 °C) δ 7.62 (dd, *J* = 8.02, 4.58 Hz, 1H), 7.51 (td, *J* = 8.59, 2.29 Hz, 1H), 7.51 (td, *J* = 8.59, 2.29 Hz, 1H), 7.39 (dd, *J* = 7.45, 2.29 Hz, 1H), 4.15-3.89 (m, 5H), 3.59 (d, *J* = 16 Hz, 1H), 1.35 (s, 9H), 1.19-1.03 (m, 6H); ^{13}C NMR (125 MHz, DMSO-*d6*, 100 °C) δ 192.54, 161.38 (d, *J* = 245.7 Hz, H), 155.66, 154.55, 134.7 (d, *J* = 7.3 Hz, H), 127.76 (d, *J* = 7.3 Hz, H), 122.59 (d, *J* = 23.2 Hz, H), 109.1 (d, *J* = 22.0 Hz, H), 82.11, 61.73, 60.45, 26.79, 13.59, 13.53; HRMS (ESI, M+Na) calcd for 447.1543, found 447.1520.

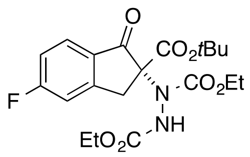

5h: $[\alpha]^{25}_{D}$ = −77.1 (*c* 4.34, $CHCl_3$, 86% ee); HPLC analysis: Daicel Chiralpak AD-H, hexane/2-propanol = 90:10, flow rate = 1.0 mL/min, τ_1 (major) = 12.5, τ_2 (minor) = 14.4 ^{1}H NMR (500 MHz, DMSO-*d6*, 100 °C) δ 7.74 (dd, *J* = 8.26, 5.16 Hz, 1H), 7.39 (d, *J* = 8.59 Hz, 1H), 7.39 (d, *J* = 8.59 Hz, 1H), 7.23 (t, *J* = 8.59 Hz, 1H), 4.16-3.90 (m, 5H), 3.65 (d, *J* = 17.5 Hz, 1H), 1.36 (s, 9H), 1.17 (br, 3H), 1.09 (br, 3H); ^{13}C NMR (125 MHz, DMSO-*d6*, 100 °C) δ 191.53, 166.52 (d, *J* = 255.5 Hz, H), 155.65, 154.58, 126.69 (d, *J* = 11 Hz, H), 115.37 (d, *J* = 24.4 Hz, H), 112.42 (d, *J* = 22 Hz, H), 81.98, 78.56, 61.69, 60.43, 26.8, 13.54; HRMS (ESI, M+Na) calcd for 447.1543, found 447.1586.

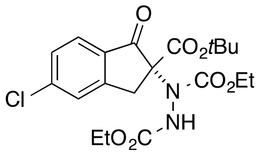

5i: $[\alpha]^{25}_{D}$ = −102.4 (*c* 4.28, $CHCl_3$, 80% ee); HPLC analysis: Daicel Chiralpak OD-H, hexane/ethanol = 99:1, flow rate = 1.0 mL/min, τ_1 (minor) = 24.5, τ_2 (major) = 27.2.

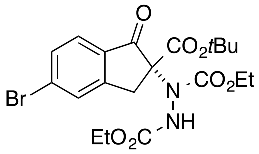

5j: $[\alpha]^{25}_{D}$ = −34.8 (*c* 4.32, $CHCl_3$, 80% ee); HPLC analysis: Daicel Chiralpak AD-H, hexane/2-propanol = 90:10, flow rate = 1.0 mL/min, τ_1 (major) = 10.8, τ_2 (minor) = 13.0; ^{1}H NMR (500 MHz, DMSO-*d6*, 100 °C) δ 7.83 (d, *J* = 9.7 Hz, 1H), 7.62-7.57 (m, 2H), 7.62-7.57 (m, 2H), 4.17-3.86 (m, 5H), 3.65 (br, 1H), 1.36 (s, 9H), 1.22-0.99 (m, 6H); ^{13}C NMR (125 MHz, DMSO-*d6*, 100 °C) δ 192.20, 155.62, 154.53, 132.10, 130.59, 129.50, 128.90, 125.24, 82.05, 61.72, 60.45, 26.80, 13.55; HRMS (ESI, M+Na) calcd for 507.0742, found 507.0786.

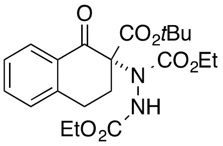

5k: $[\alpha]^{25}_{D}$ = +0.42 (*c* 3.27, $CHCl_3$, 61% ee); HPLC analysis: Daicel Chiralpak OD-H, hexane/2-propanol = 90:10, flow rate = 1.0 mL/min, τ_1 (minor) = 7.3, τ_2 (major) = 11.1.

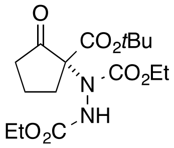

5l: $[\alpha]^{25}_{D}$ = +1.1 (*c* 2.14, $CHCl_3$, 38% ee); HPLC analysis: Daicel Chiralpak AD-H, hexane/2-propanol = 95:5, flow rate = 1.0 mL/min, τ_1 (minor) = 7.9, τ_2 (major) = 11.6.

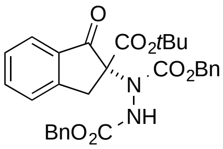

6a: $[\alpha]^{25}_{D}$ = −52.3 (*c* 4.62, $CHCl_3$, 64% ee); HPLC analysis: Daicel Chiralpak OD-H, hexane/2-propanol = 95:5, flow rate = 1.0 mL/min, τ_1 (minor) = 20.1, τ_2 (major) = 23.2.

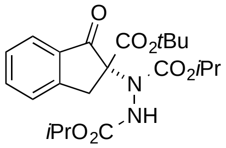

7a: $[\alpha]^{25}_{D}$ = −88.4 (*c* 4.17, $CHCl_3$, 79% ee); HPLC analysis: Daicel Chiralpak OD-H, hexane/2-propanol = 98:2, flow rate = 1.0 mL/min, τ_1 (minor) = 8.4, τ□ (major) = 9.4.

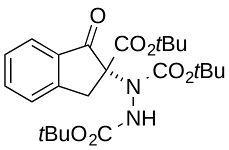

8a: $[\alpha]^{25}_{D}$ = −45.3 (*c* 2.05, $CHCl_3$, 44% ee); HPLC analysis: Daicel Chiralpak AD-H, hexane/ethanol = 95:5, flow rate = 1.0 mL/min, τ_1 (minor) = 8.7, τ_2 (major) = 13.7.

4. Chiral HPLC analysis

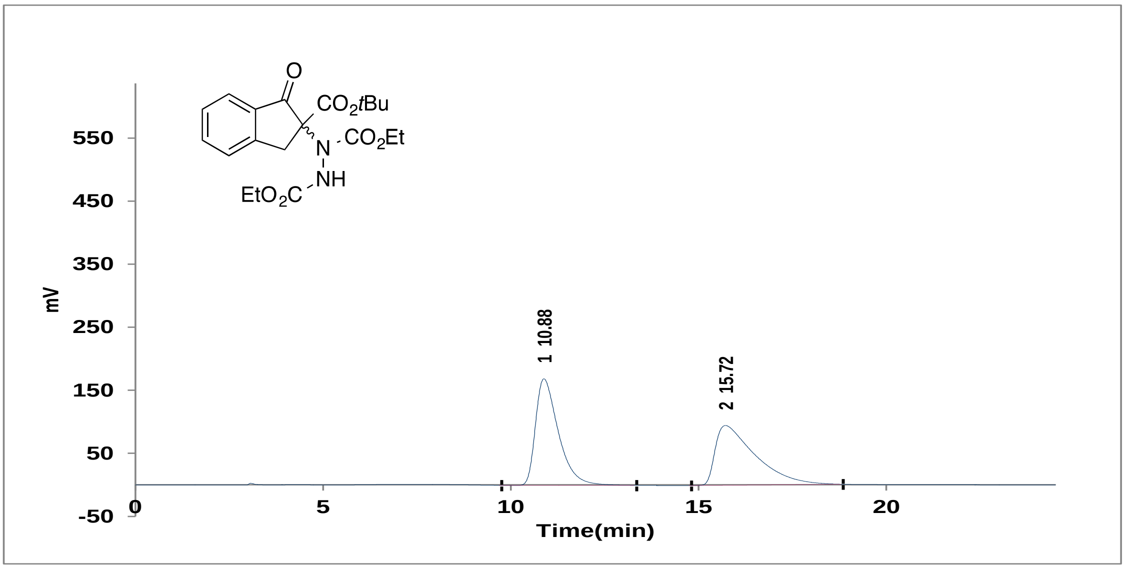

No.	Rt	Area	Area(%)	Height
1	10.88	7154192	50.5456	169192
2	15.72	6999750	49.4544	94857
		14153942	100	264049

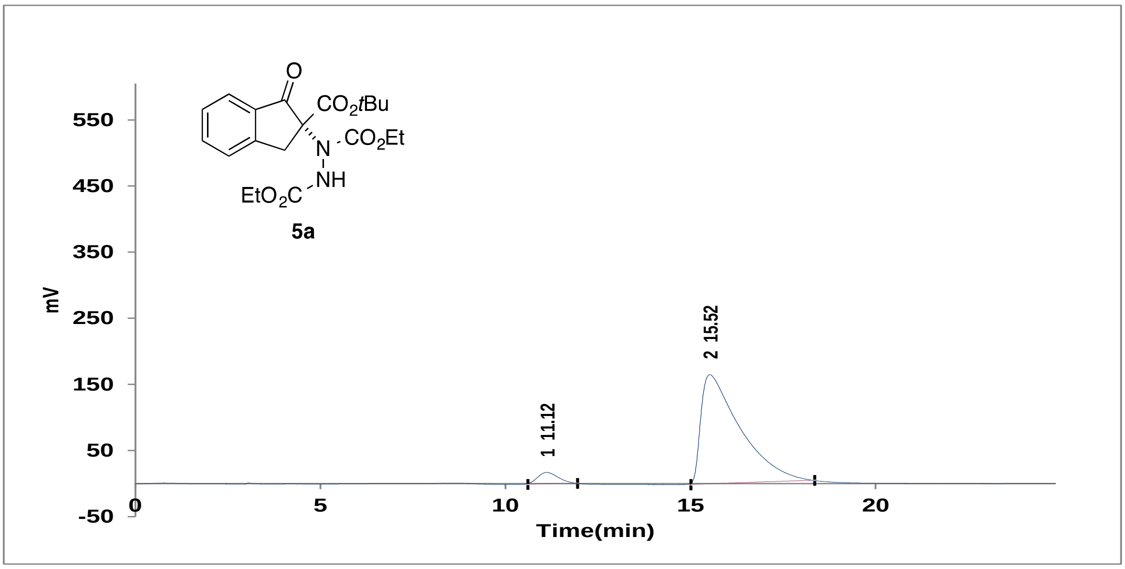

No.	Rt	Area	Area(%)	Height
1	11.12	649968.4	5.0856	17075
2	15.52	12130522	94.9144	164947
		12780490	100	182022

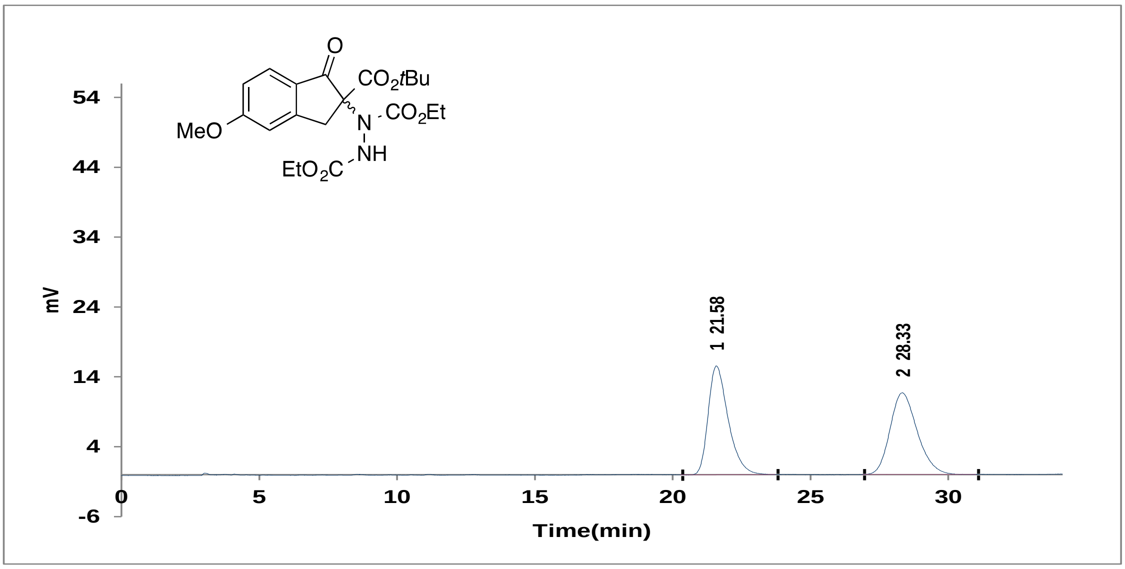

No.	Rt	Area	Area(%)	Height
1	21.58	787278.6	49.5577	15584
2	28.33	801331.9	50.4423	11703
		1588611	100	27287

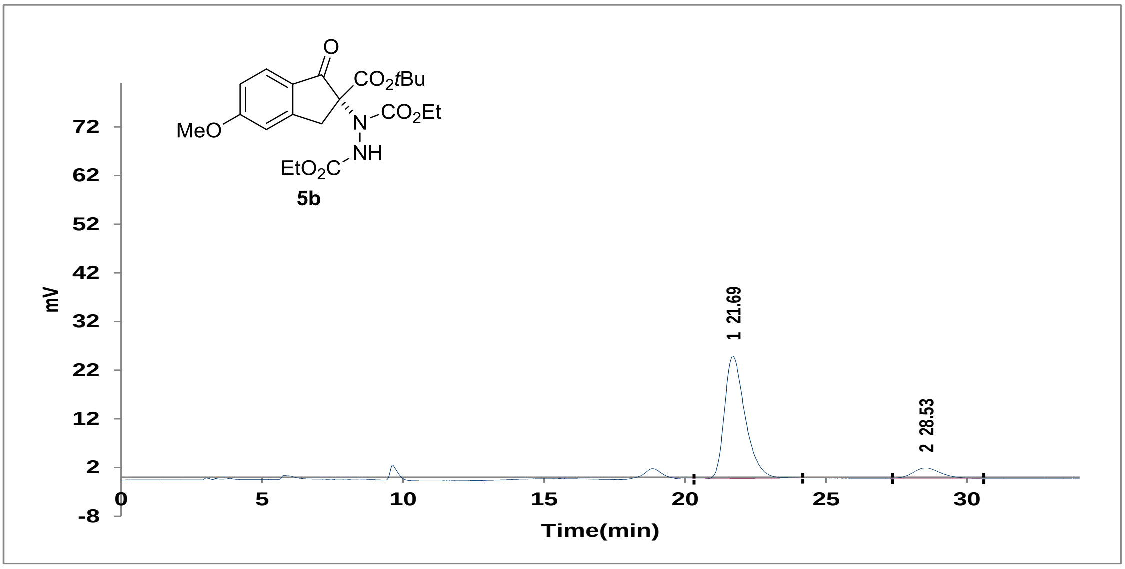

No.	Rt	Area	Area(%)	Height
1	21.69	1276703	89.9292	25193
2	28.53	142972.1	10.0708	2127
		1419675	100	27320

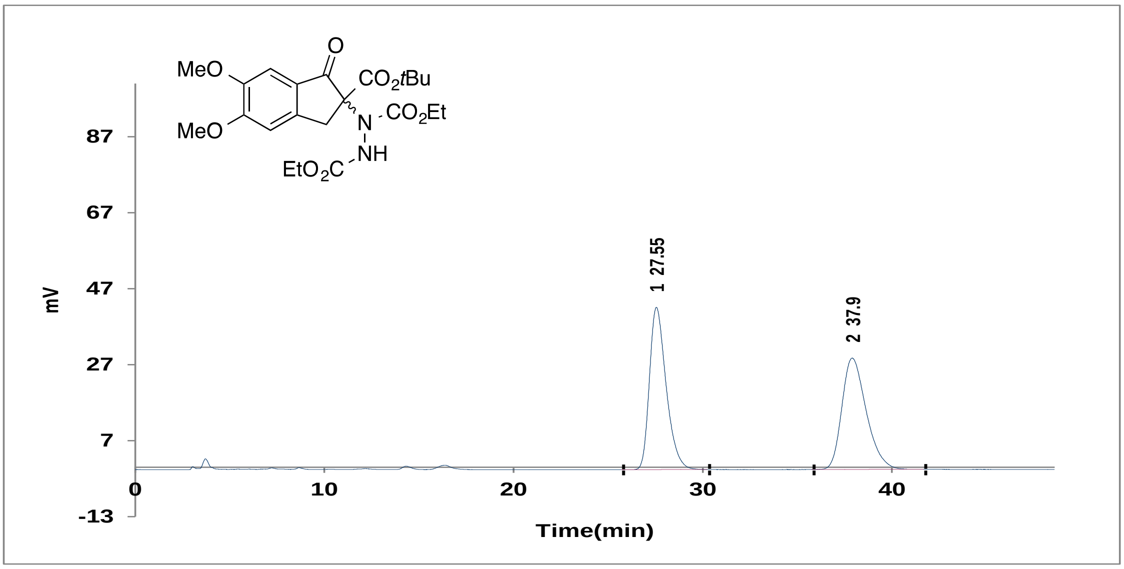

No.	Rt	Area	Area(%)	Height
1	27.55	2622756	50.0481	42729
2	37.9	2617710	49.9519	29321
		5240465	100	72050

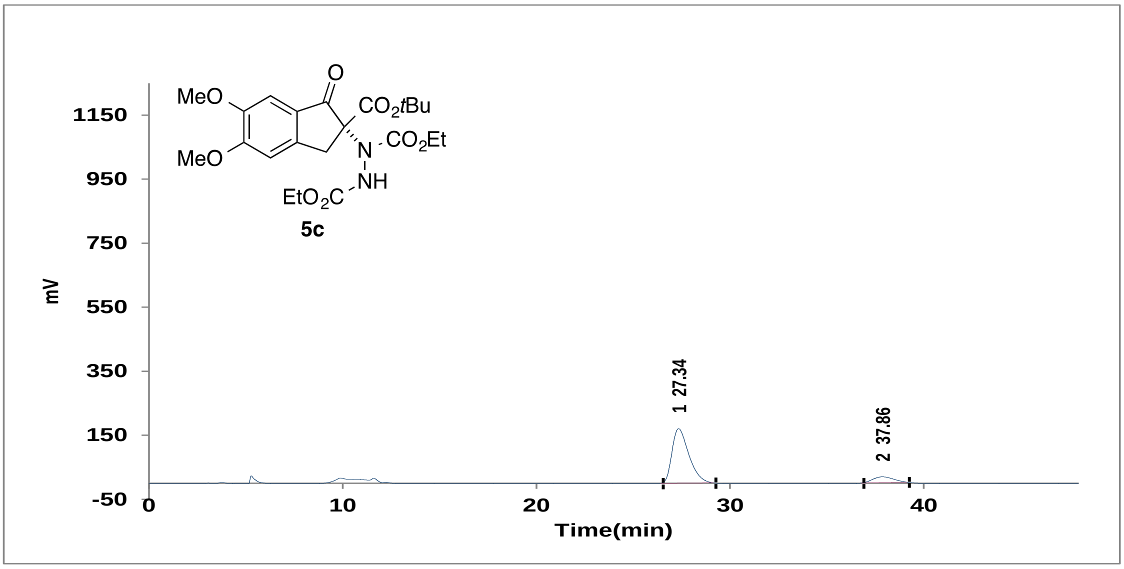

No.	Rt	Area	Area(%)	Height
1	27.34	10247155	88.5274	170219
2	37.86	1327962	11.4726	18196
		11575117	100	188415

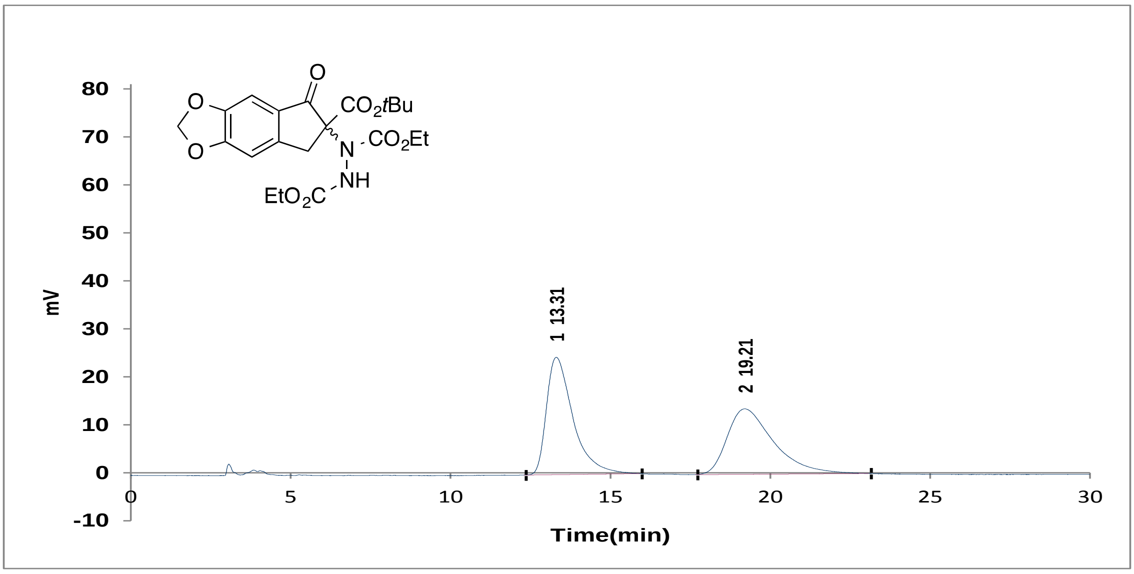

No.	Rt	Area	Area(%)	Height
1	13.31	1413088	50.6118	24469
2	19.21	1378923	49.3882	13653
		2792011	100	38122

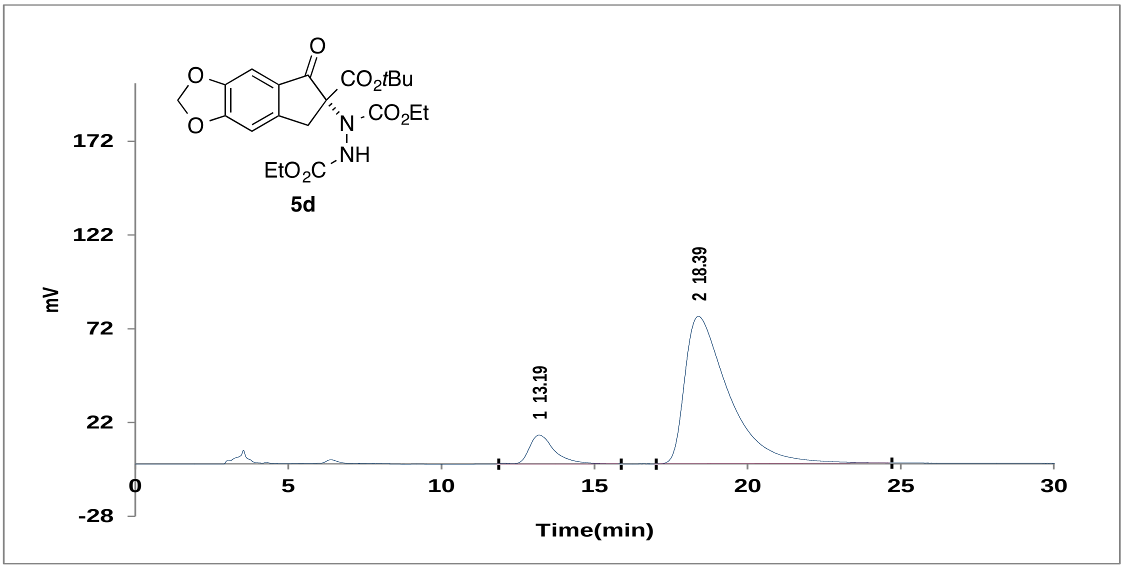

No.	Rt	Area	Area(%)	Height
1	13.19	889911.4	10.2251	15423
2	18.39	7813291	89.7749	78610
		8703203	100	94033

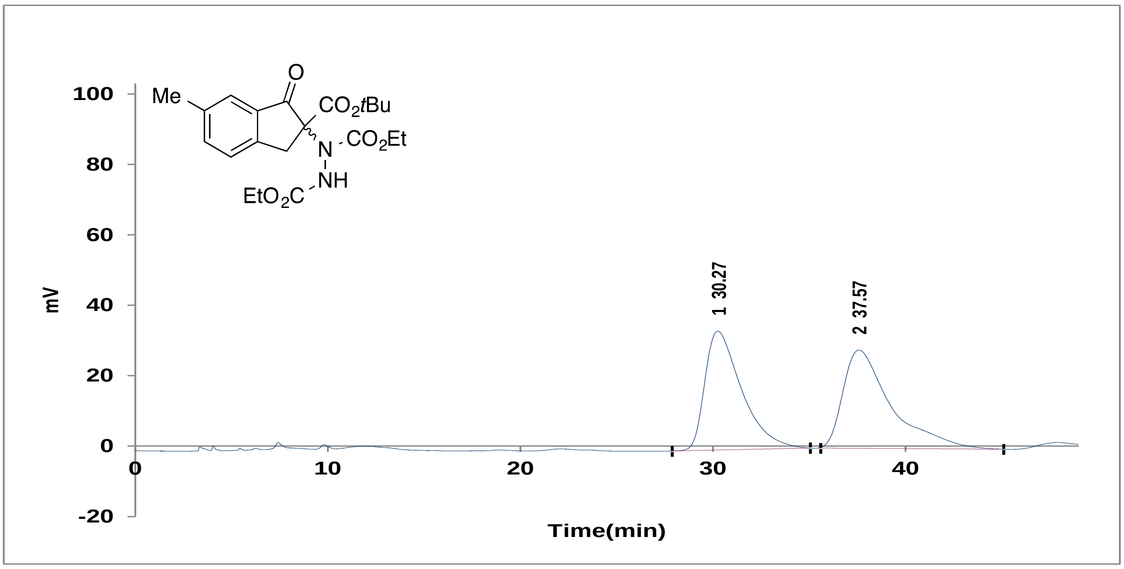

No.	Rt	Area	Area(%)	Height
1	30.27	4478031	48.0885	33749
2	37.57	4834029	51.9115	27904
		9312060	100	61653

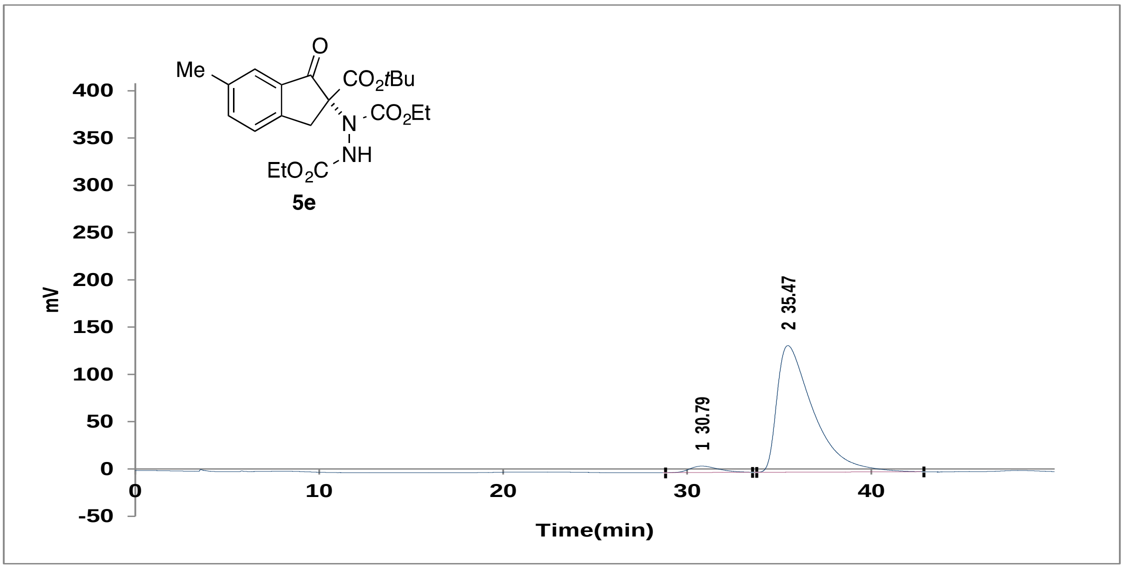

No.	Rt	Area	Area(%)	Height
1	30.79	725378.2	3.7986	6767
2	35.47	18370579	96.2014	133922
		19095957	100	140689

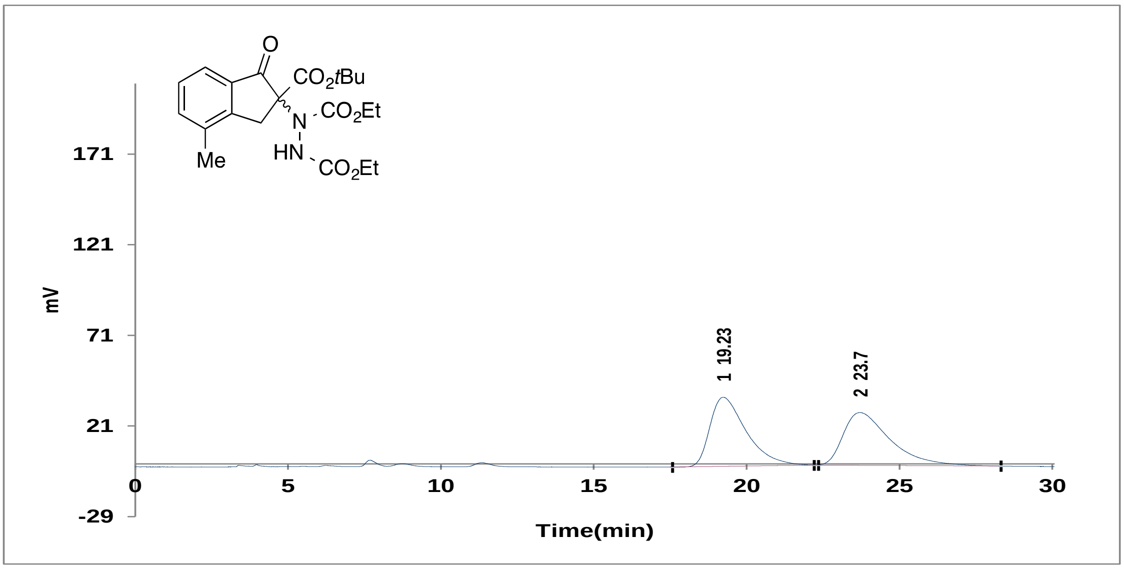

No.	Rt	Area	Area(%)	Height
1	19.23	3139235	50.2216	38152
2	23.7	3111526	49.7784	29153
		6250761	100	67305

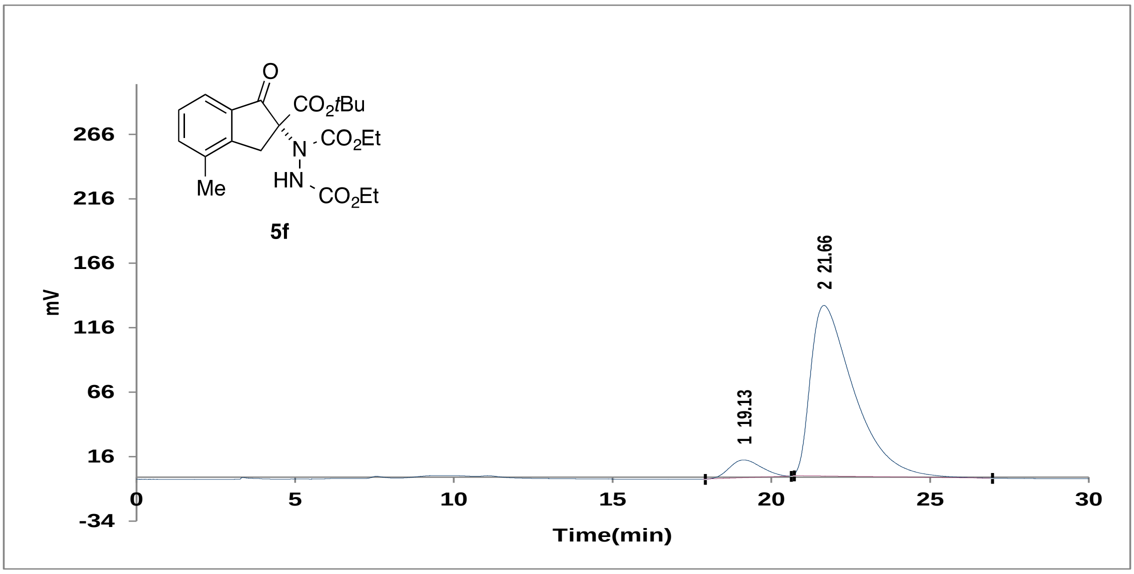

No.	Rt	Area	Area(%)	Height
1	19.13	955852.7	7.056	13747
2	21.66	12590880	92.944	132302
		13546733	100	146049

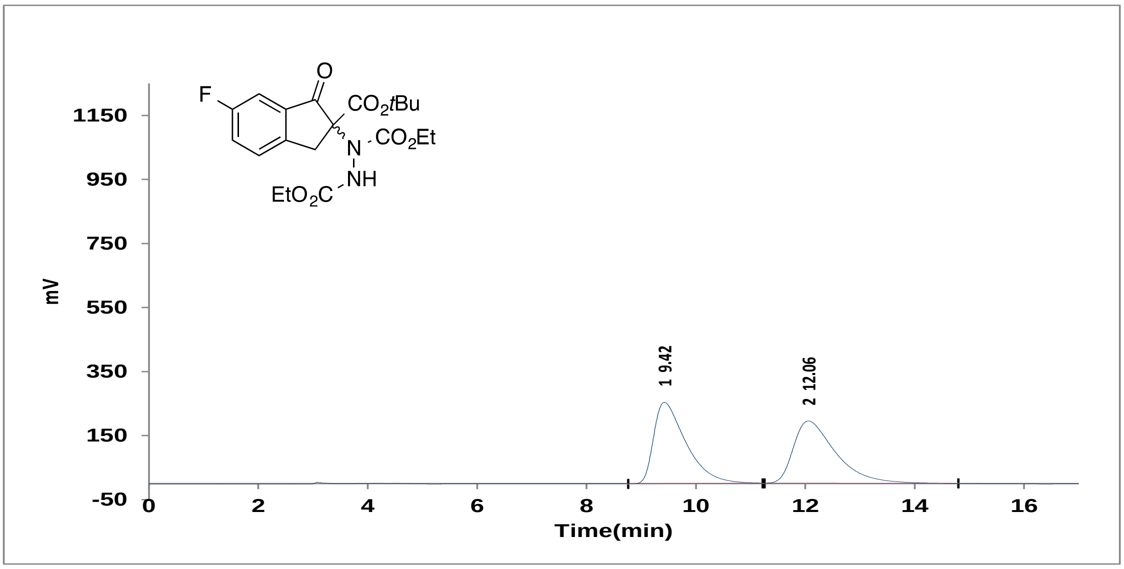

No.	Rt	Area	Area(%)	Height
1	9.84	650706.8	12.5099	15318
2	12.17	4550817	87.4901	71997
		5201524	100	87315

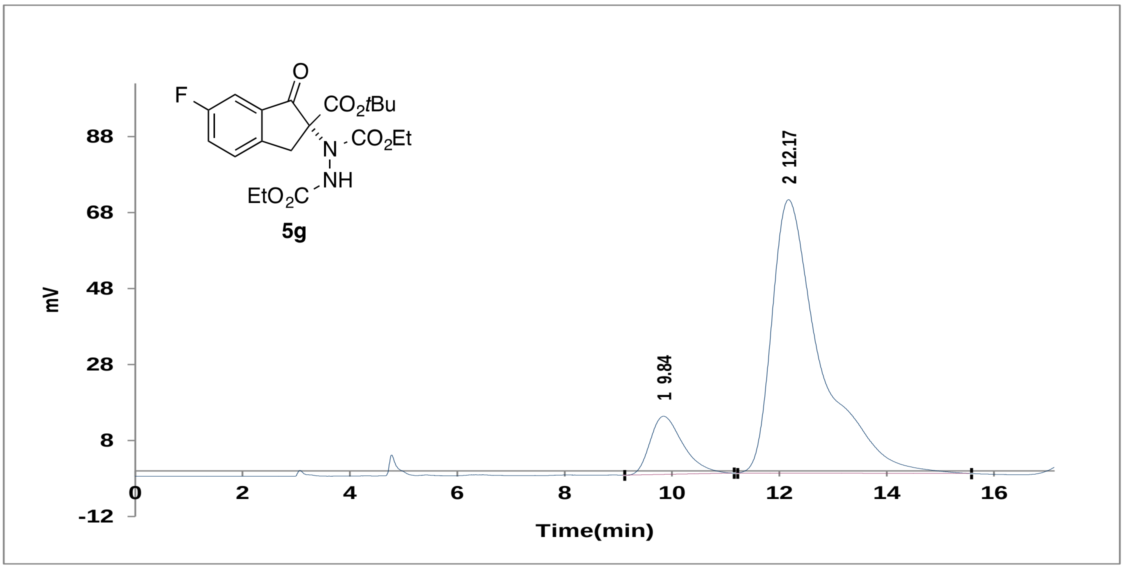

No.	Rt	Area	Area(%)	Height
1	9.42	10418790	49.969	254206
2	12.06	10431716	50.031	194350
		20850506	100	448556

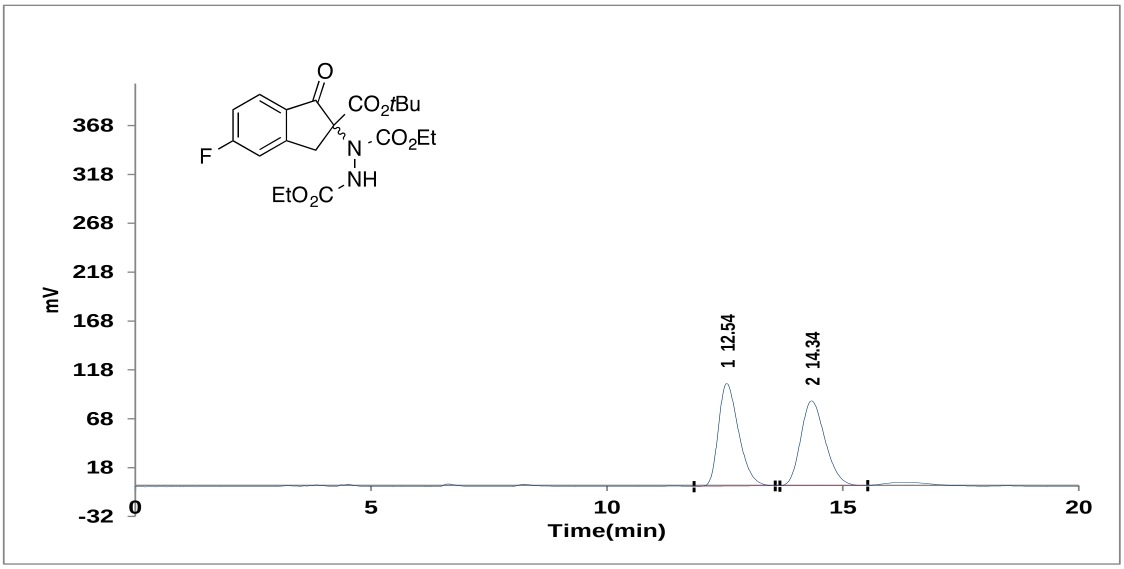

No.	Rt	Area	Area(%)	Height
1	12.54	3128289	50.0666	104900
2	14.34	3119969	49.9334	86807
		6248258	100	191707

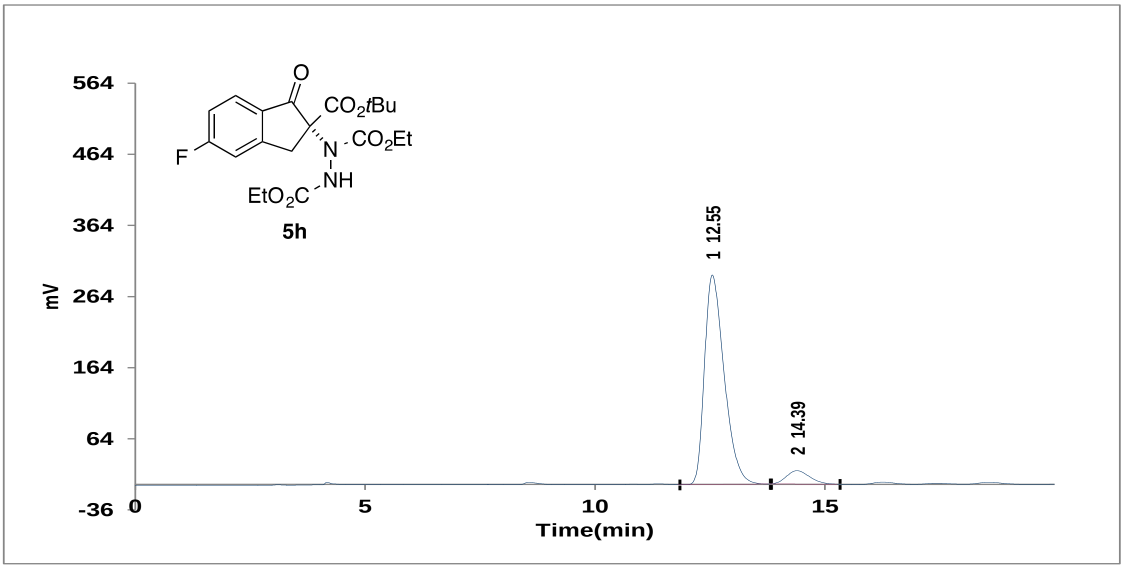

No.	Rt	Area	Area(%)	Height
1	12.55	8748736	92.9766	294359
2	14.39	660879.2	7.0234	18766
		9409615	100	313125

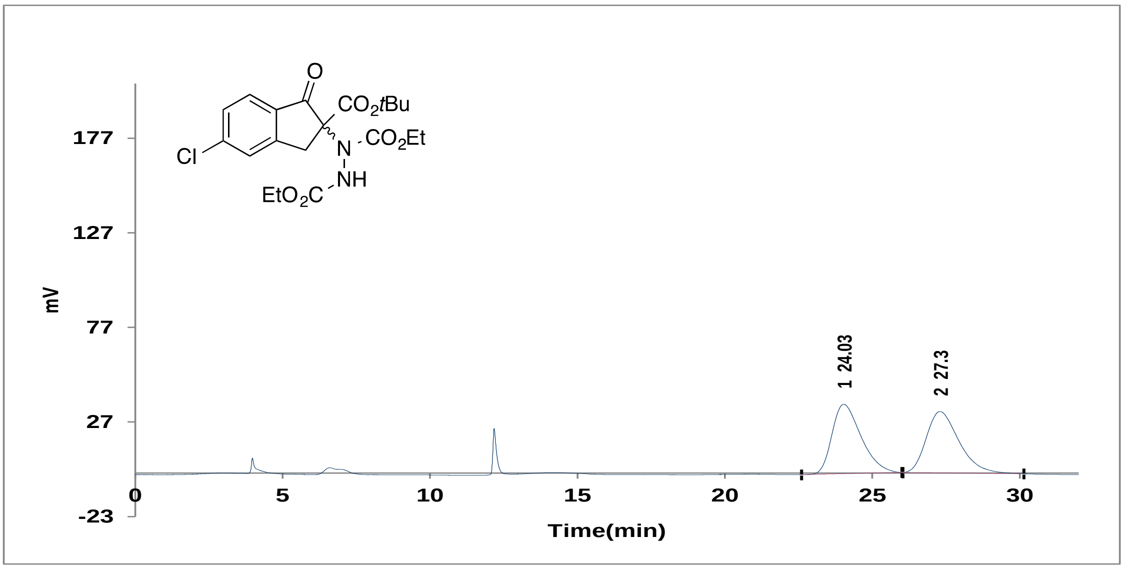

No.	Rt	Area	Area(%)	Height
1	24.03	2508261	50.0499	36714
2	27.3	2503260	49.9501	32395
		5011521	100	69109

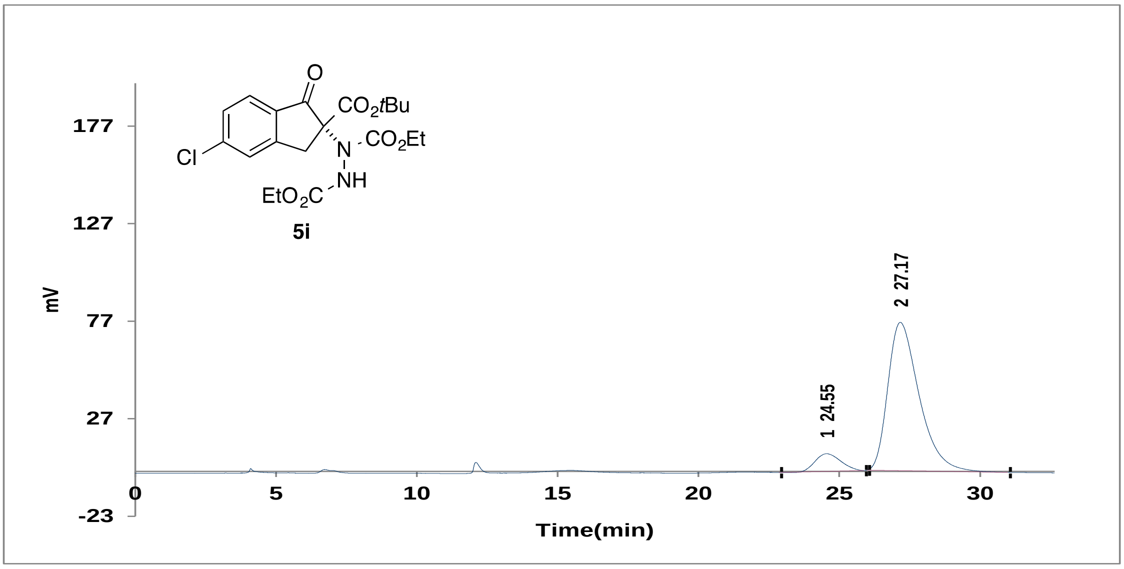

No.	Rt	Area	Area(%)	Height
1	24.55	566194.9	8.7922	9046
2	27.17	5873580	91.2078	76047
		6439775	100	85093

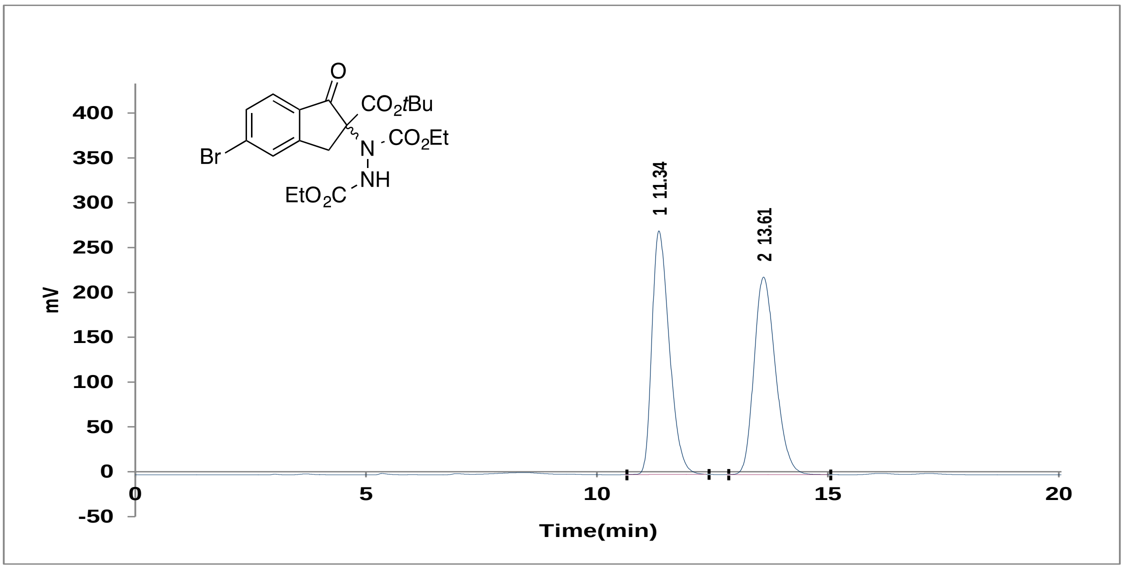

No.	Rt	Area	Area(%)	Height
1	11.34	7124806	50.0097	271745
2	13.61	7122039	49.9903	220337
		14246845	100	492082

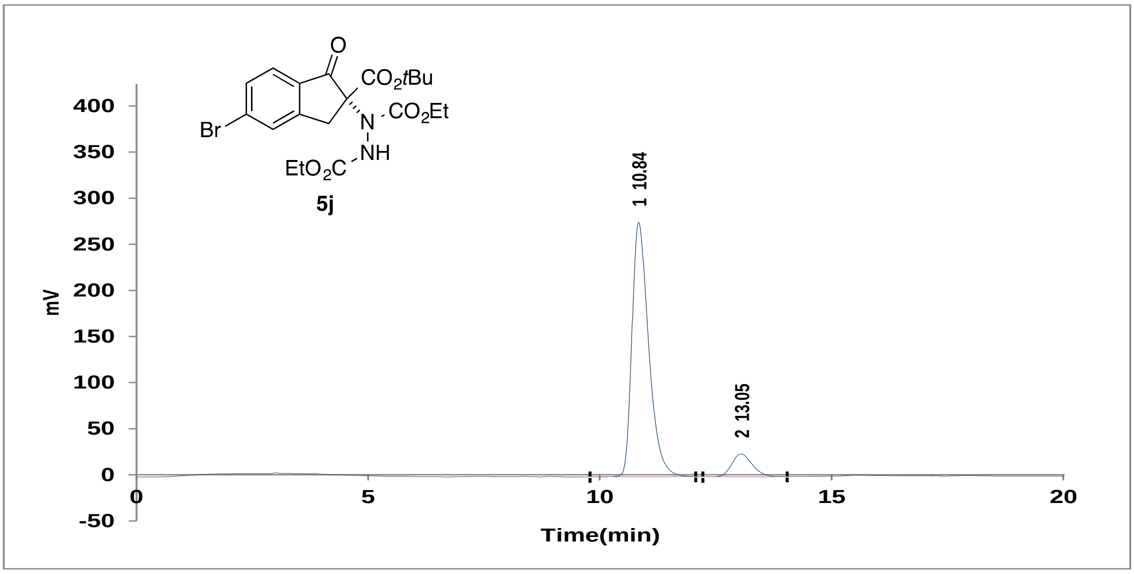

No.	Rt	Area	Area(%)	Height
1	10.84	6933706	90.1684	275952
2	13.05	756021.4	9.8316	24754
		7689728	100	300706

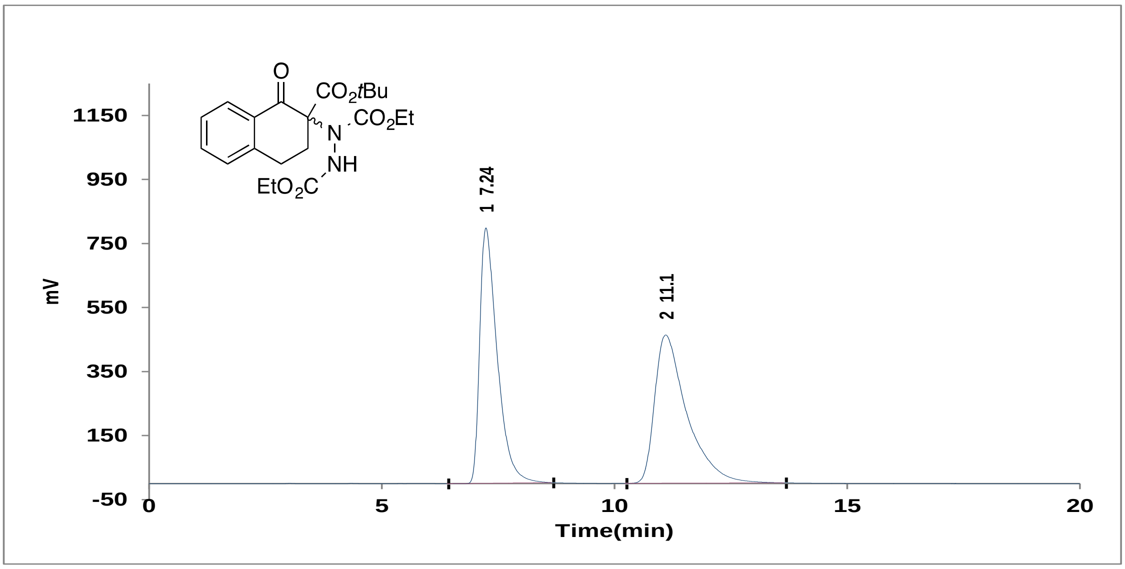

No.	Rt	Area	Area(%)	Height
1	7.24	20813954	48.347	798060
2	11.1	22237189	51.653	463385
		43051143	100	1261445

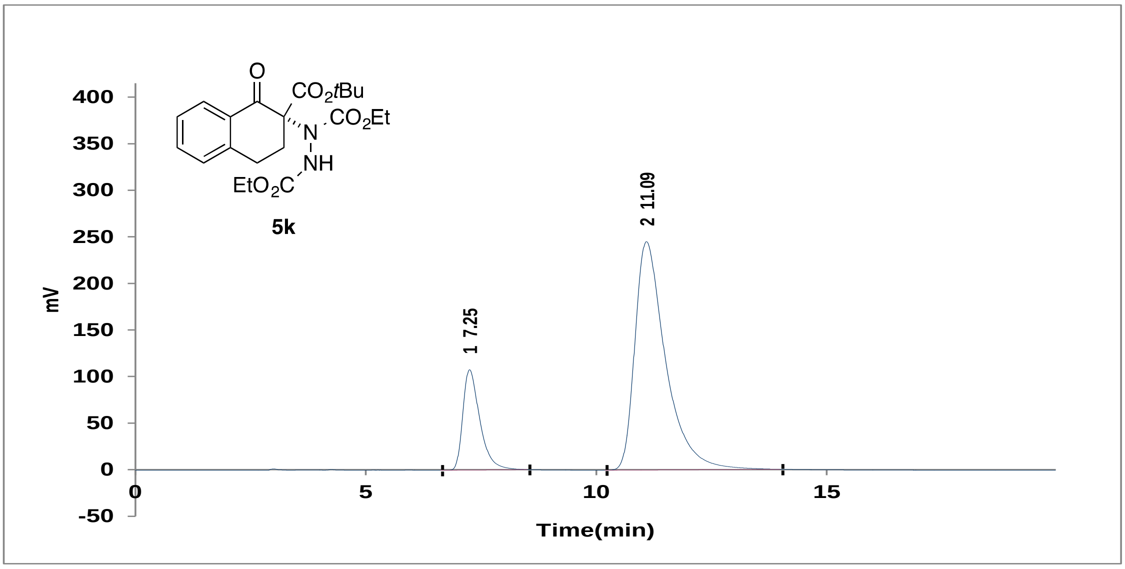

No.	Rt	Area	Area(%)	Height
1	7.25	2769507	19.5392	107824
2	11.09	11404599	80.4608	245252
		14174106	100	353076

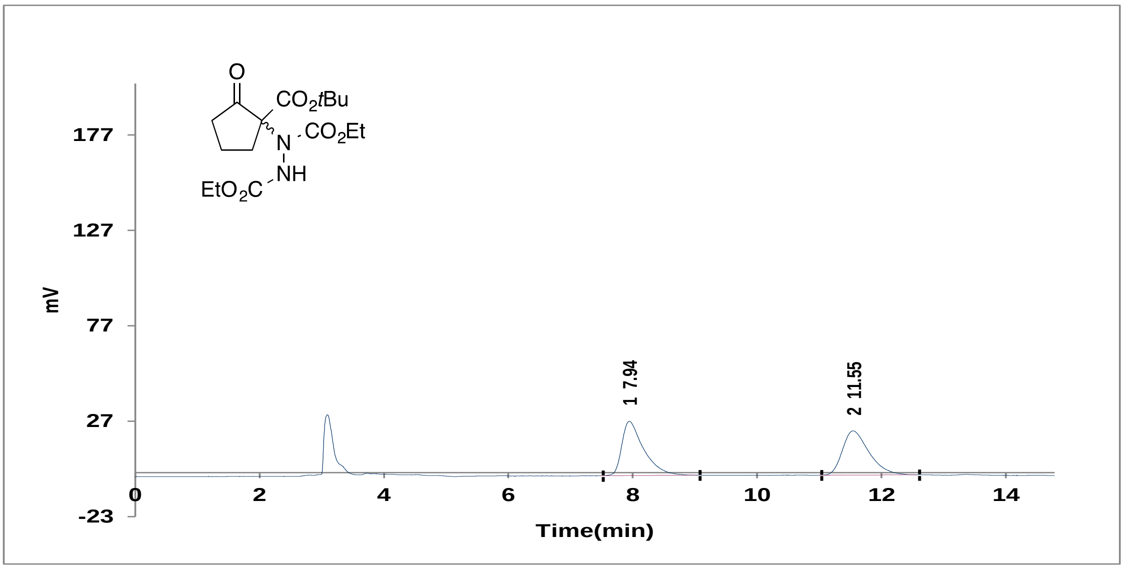

No.	Rt	Area	Area(%)	Height
1	7.94	694449.2	50.1708	28500
2	11.55	689720.6	49.8292	23174
		1384170	100	51674

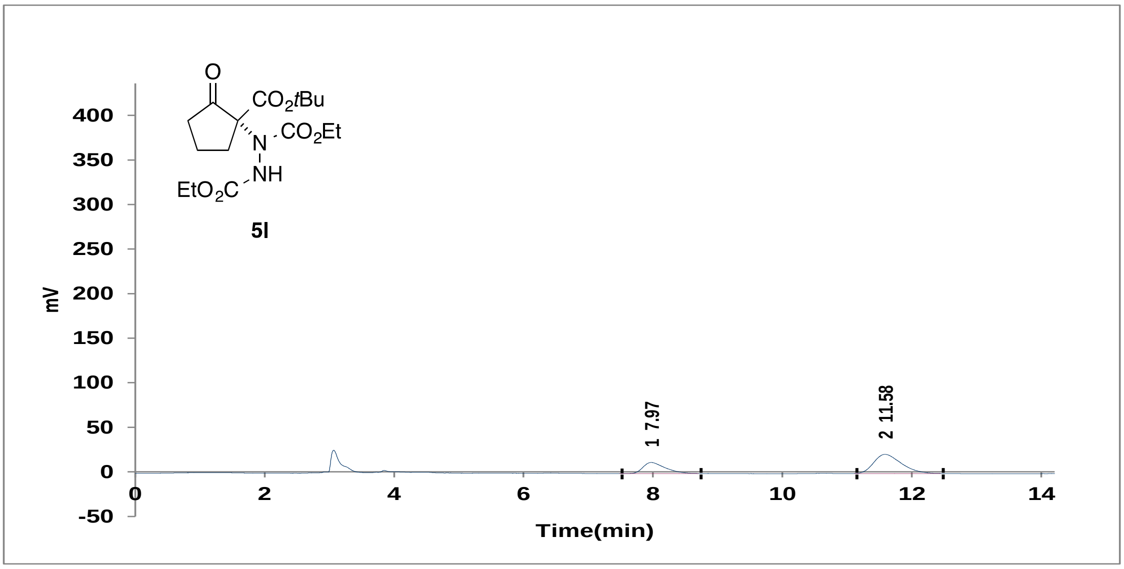

No.	Rt	Area	Area(%)	Height
1	7.97	295183.2	31.6648	12419
2	11.58	637029.3	68.3352	21472
		932212.5	100	33891

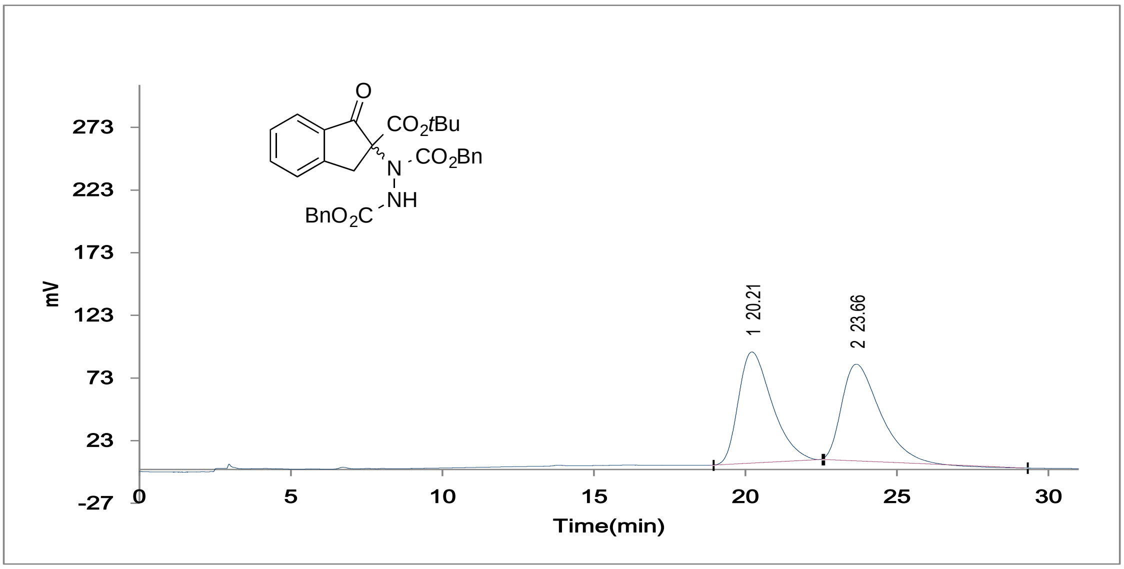

No.	Rt	Area	Area(%)	Height
1	20.21	6874927	50.3844	88722
2	23.66	6770026	49.6156	77207
		13644953	100	165929

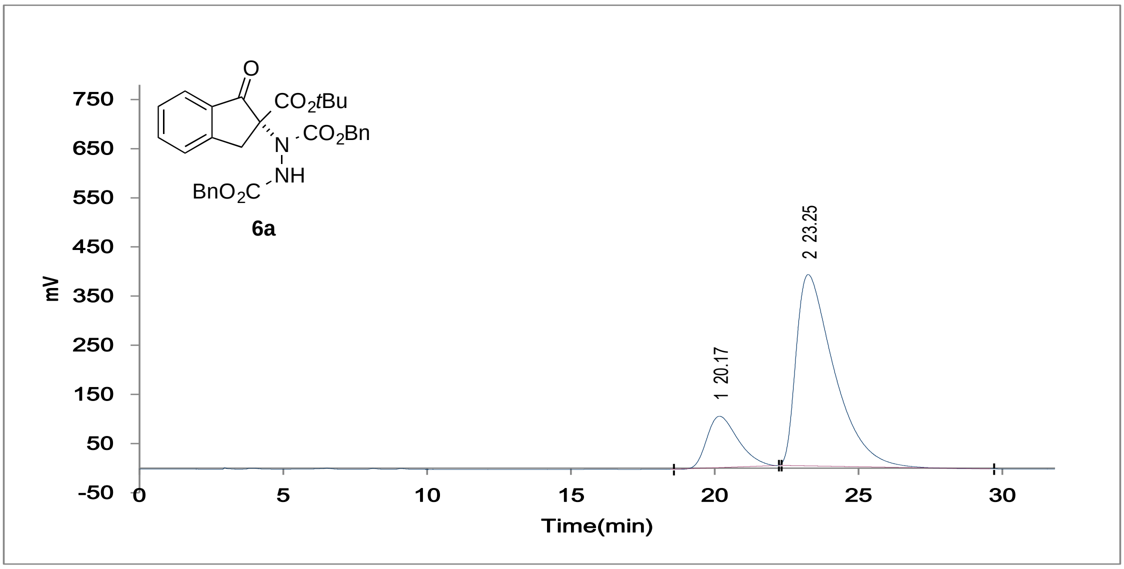

No.	Rt	Area	Area(%)	Height
1	20.17	7820545	17.9652	104472
2	23.25	35711101	82.0348	389152
		43531646	100	493624

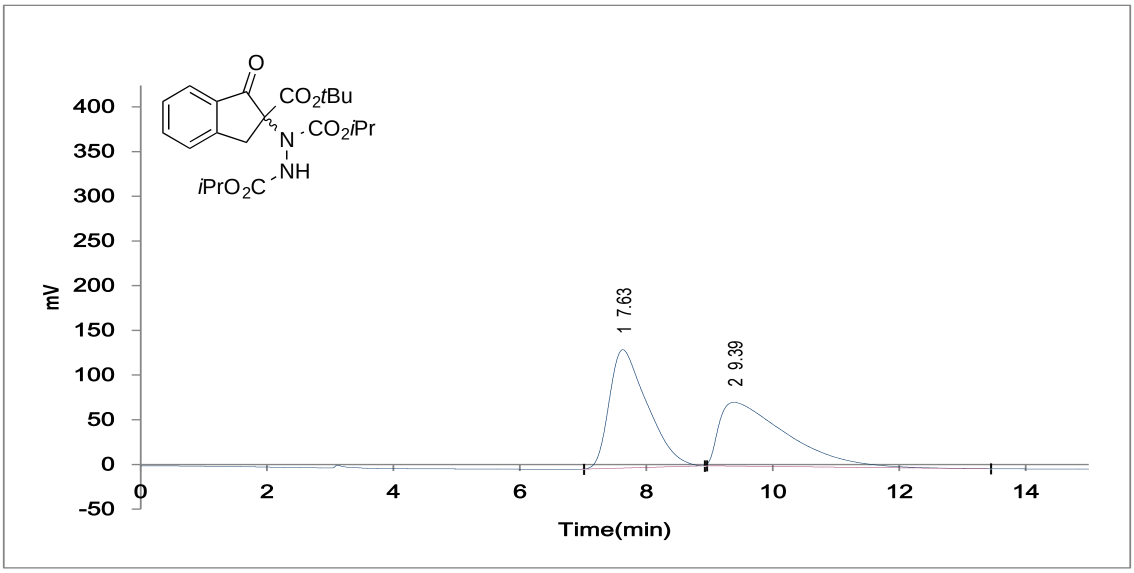

No.	Rt	Area	Area(%)	Height
1	7.63	5506573	51.0023	132396
2	9.39	5290147	48.9977	71312
		10796721	100	203708

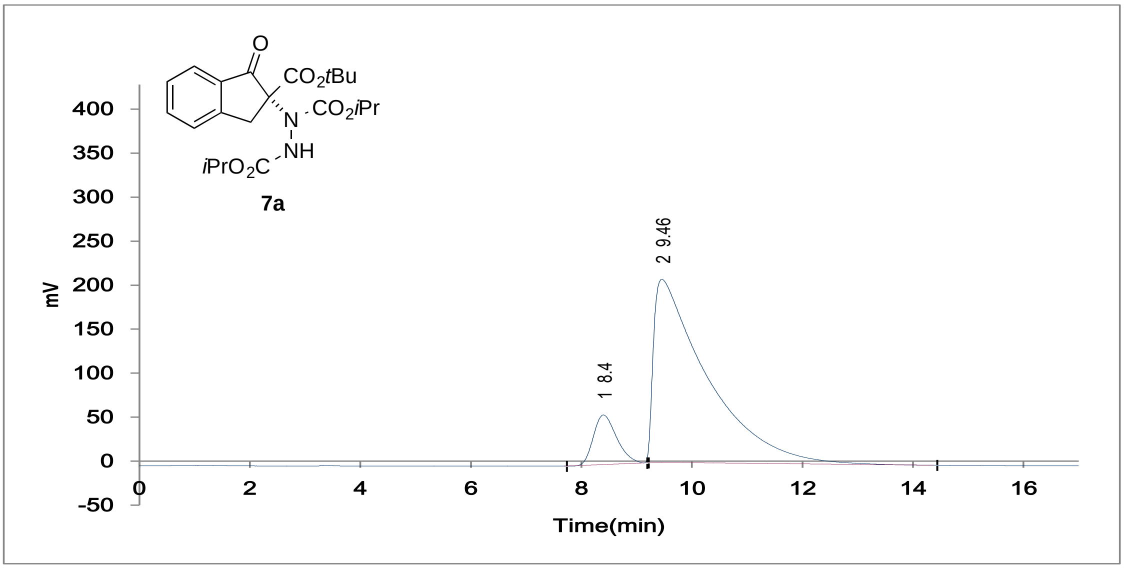

No.	Rt	Area	Area(%)	Height
1	8.4	1640928	10.509	56356
2	9.46	13973642	89.491	208102
		15614570	100	264458

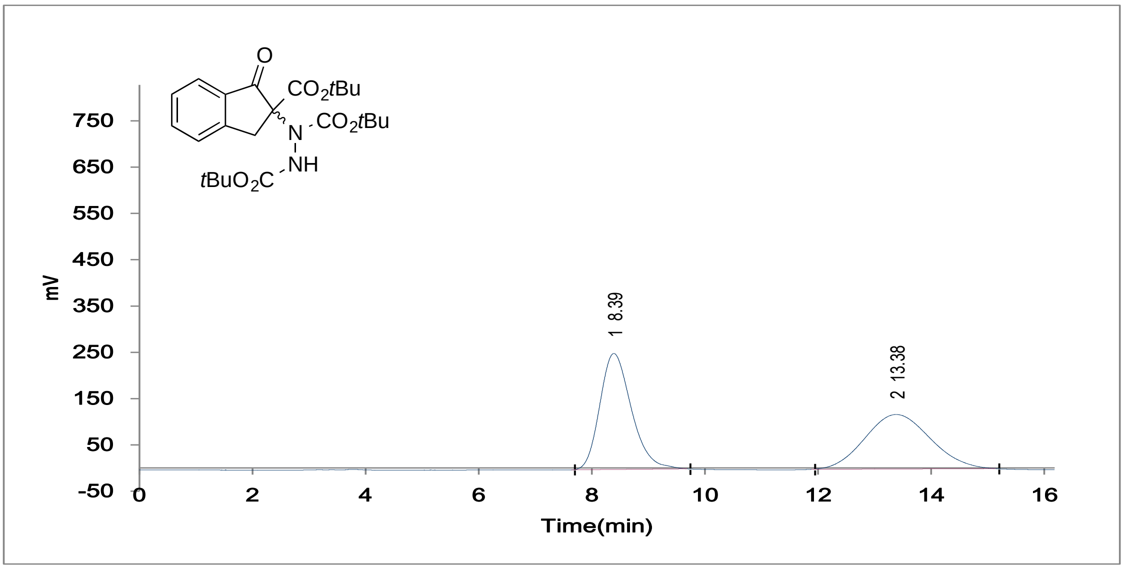

No.	Rt	Area	Area(%)	Height
1	8.39	9699897	50.5589	250744
2	13.38	9485447	49.4411	117814
		19185345	100	368558

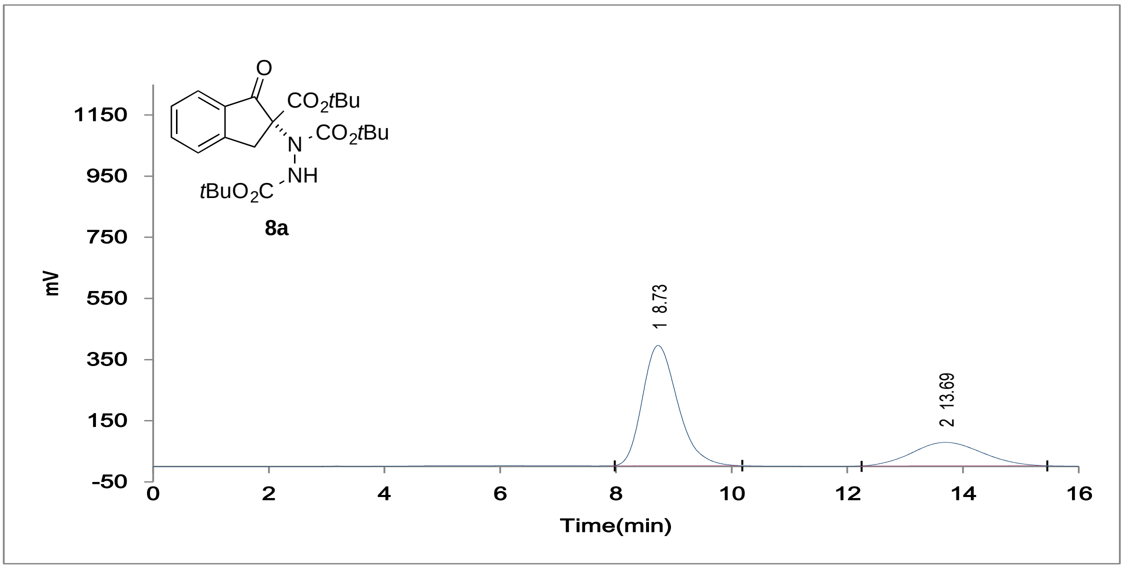

No.	Rt	Area	Area(%)	Height
1	8.73	16624336	72.0583	393342
2	13.69	6446350	27.9417	76819
		23070685	100	470161

5. ^{1}H and ^{13}C NMR spectra of new compounds 5d–h, 5j

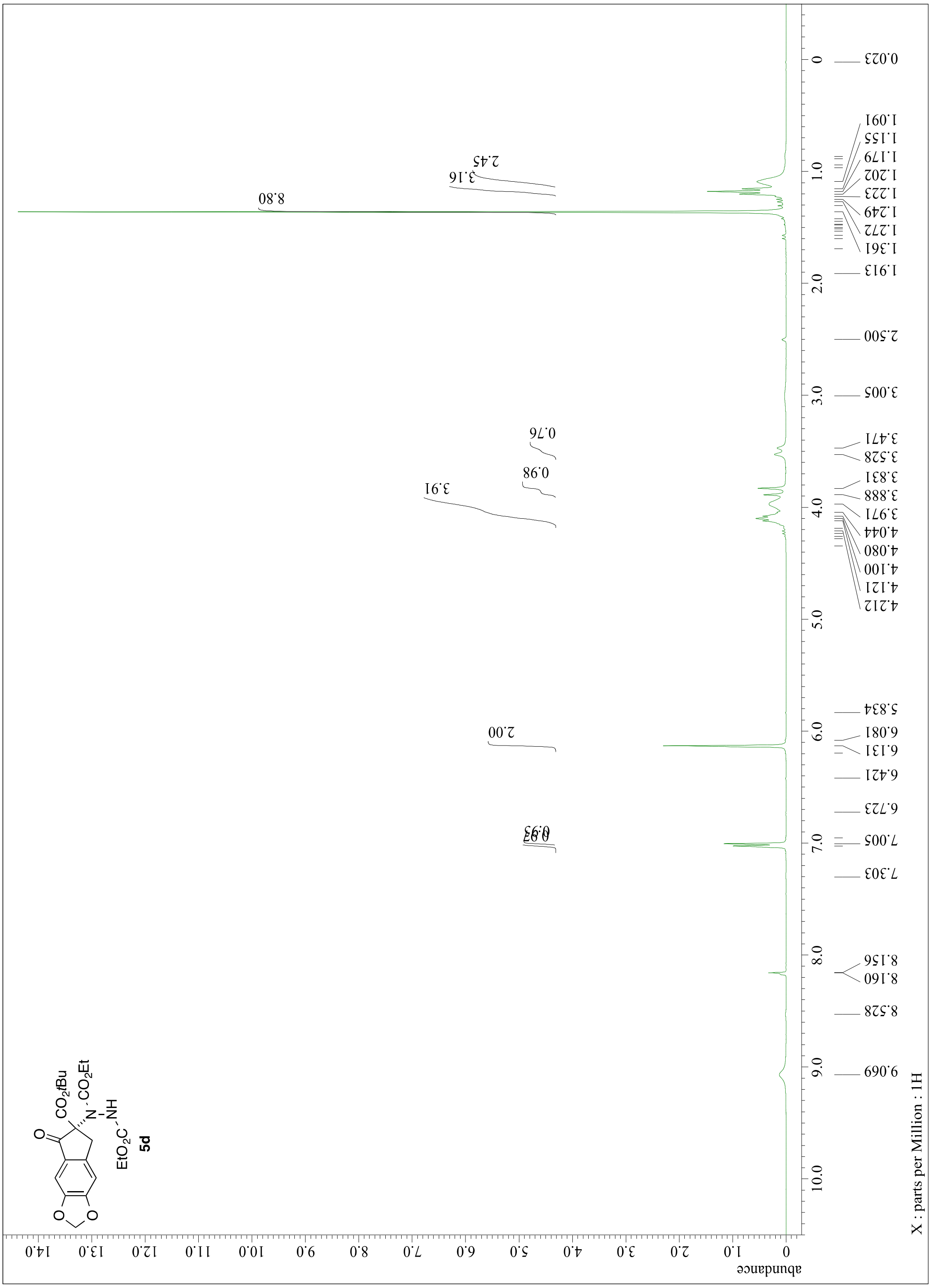

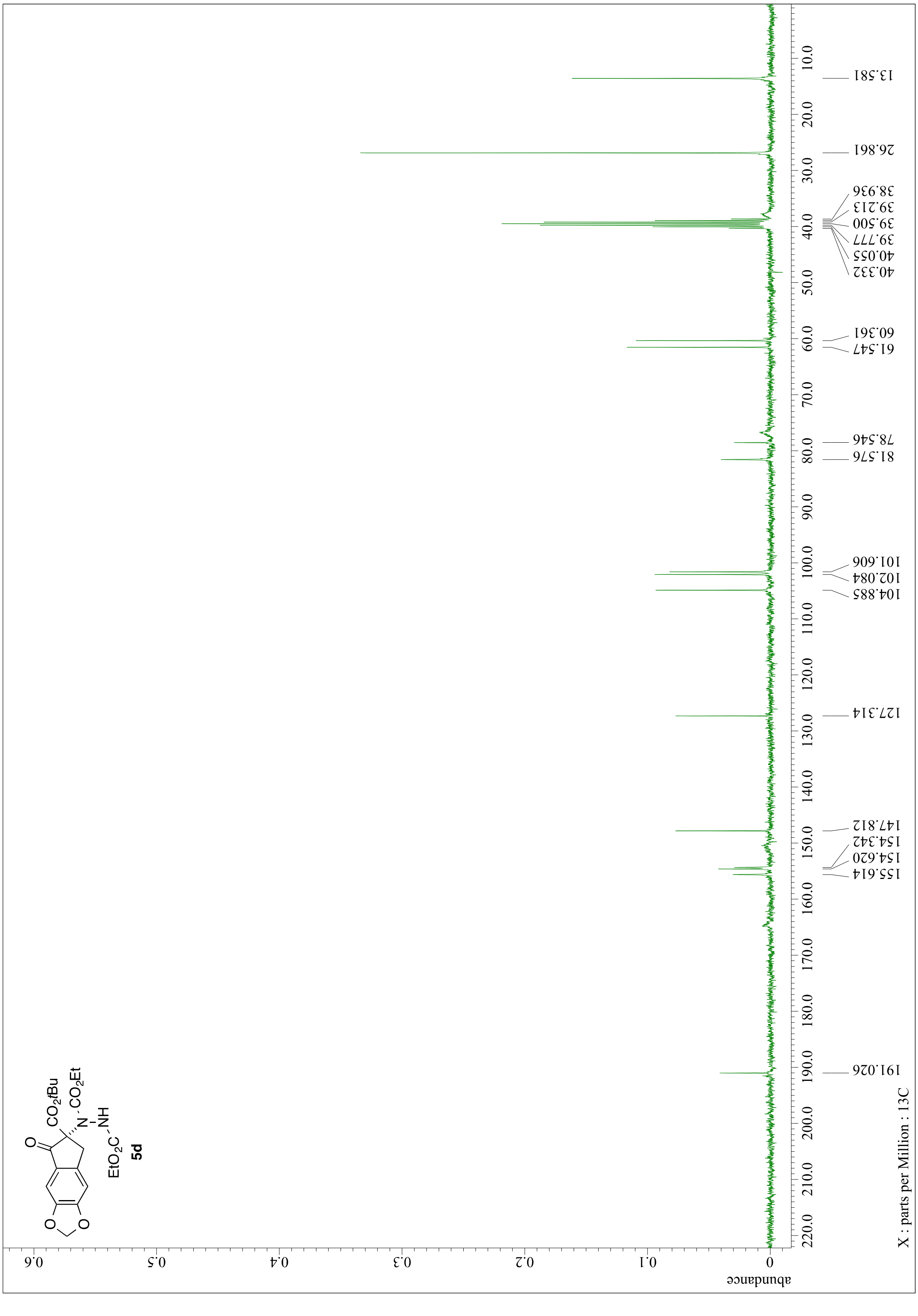

13.581
26.861
38.936
39.213
39.500
39.777
40.055
40.332
60.361
61.547
78.546
81.576
101.606
102.084
104.885
127.314
147.812
154.342
154.620
155.614
191.026
X : parts per Million : 13C
abundance
5d

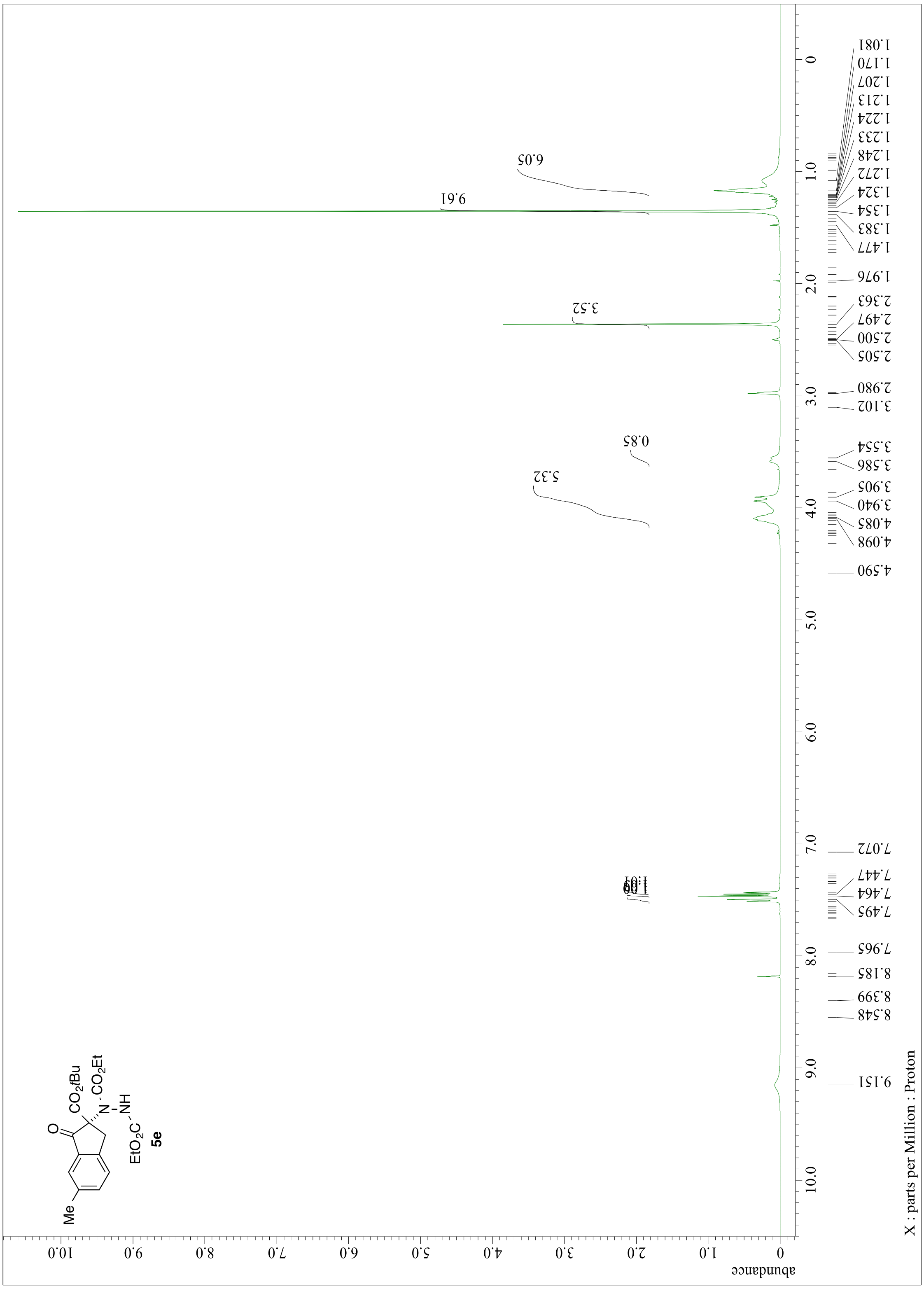
X : parts per Million : Proton
abundance
CO2tBu
N
CO2Et
NH
EtO2C
Me
O
5e
1.081
1.170
1.207
1.213
1.224
1.233
1.248
1.272
1.324
1.354
1.383
1.477
1.976
2.363
2.497
2.500
2.505
2.980
3.102
3.554
3.586
3.905
3.940
4.085
4.098
4.590
7.072
7.447
7.464
7.495
7.965
8.185
8.399
8.548
9.151
6.05
9.61
3.52
0.85
5.32
1.00
1.01

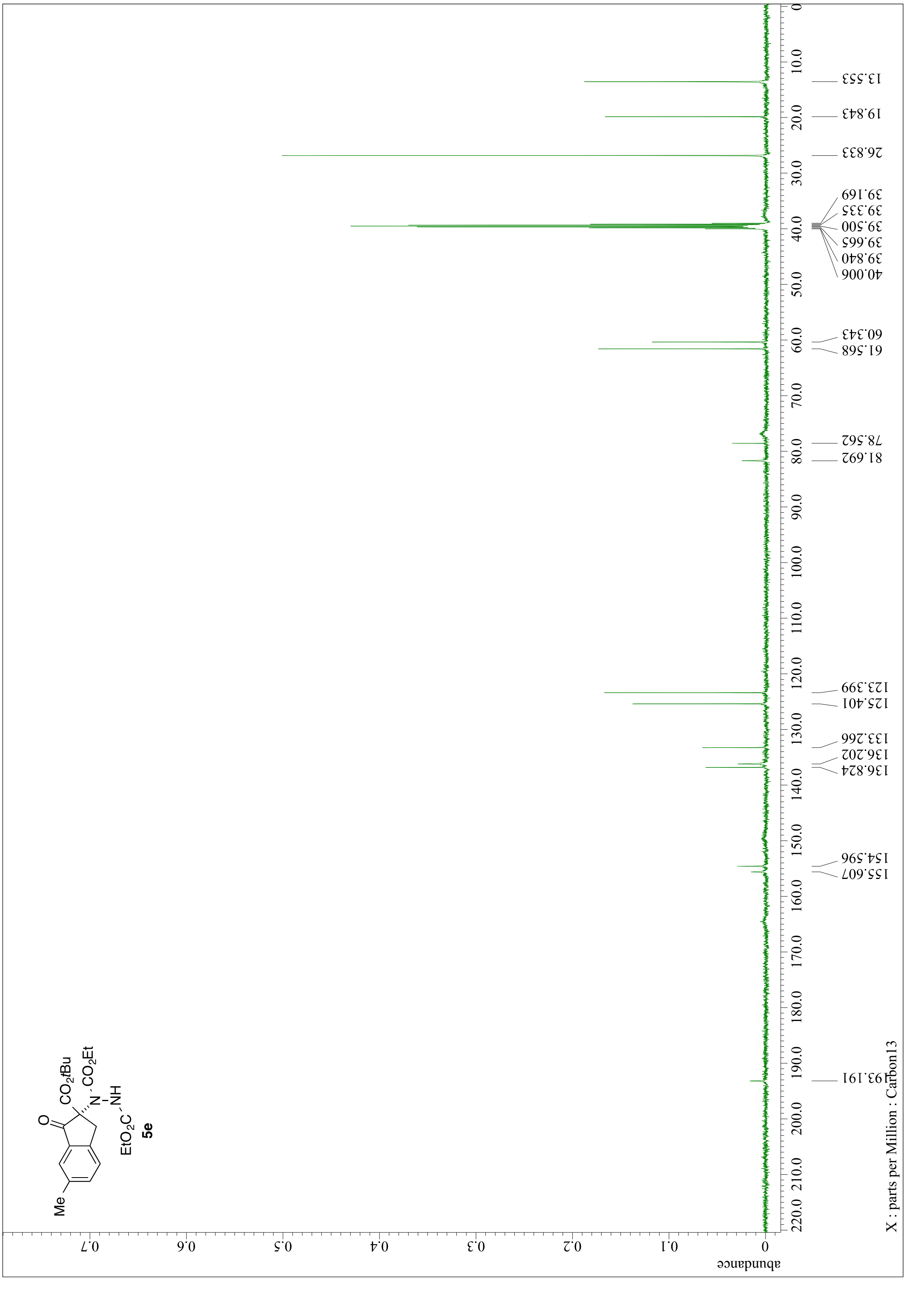
Me
CO2tBu
N
CO2Et
NH
EtO2C
5e
13.553
19.843
26.833
39.169
39.335
39.500
39.665
39.840
40.006
60.343
61.568
78.562
81.692
123.399
125.401
133.266
136.202
136.824
154.596
155.607
193.191
X : parts per Million : Carbon13
abundance

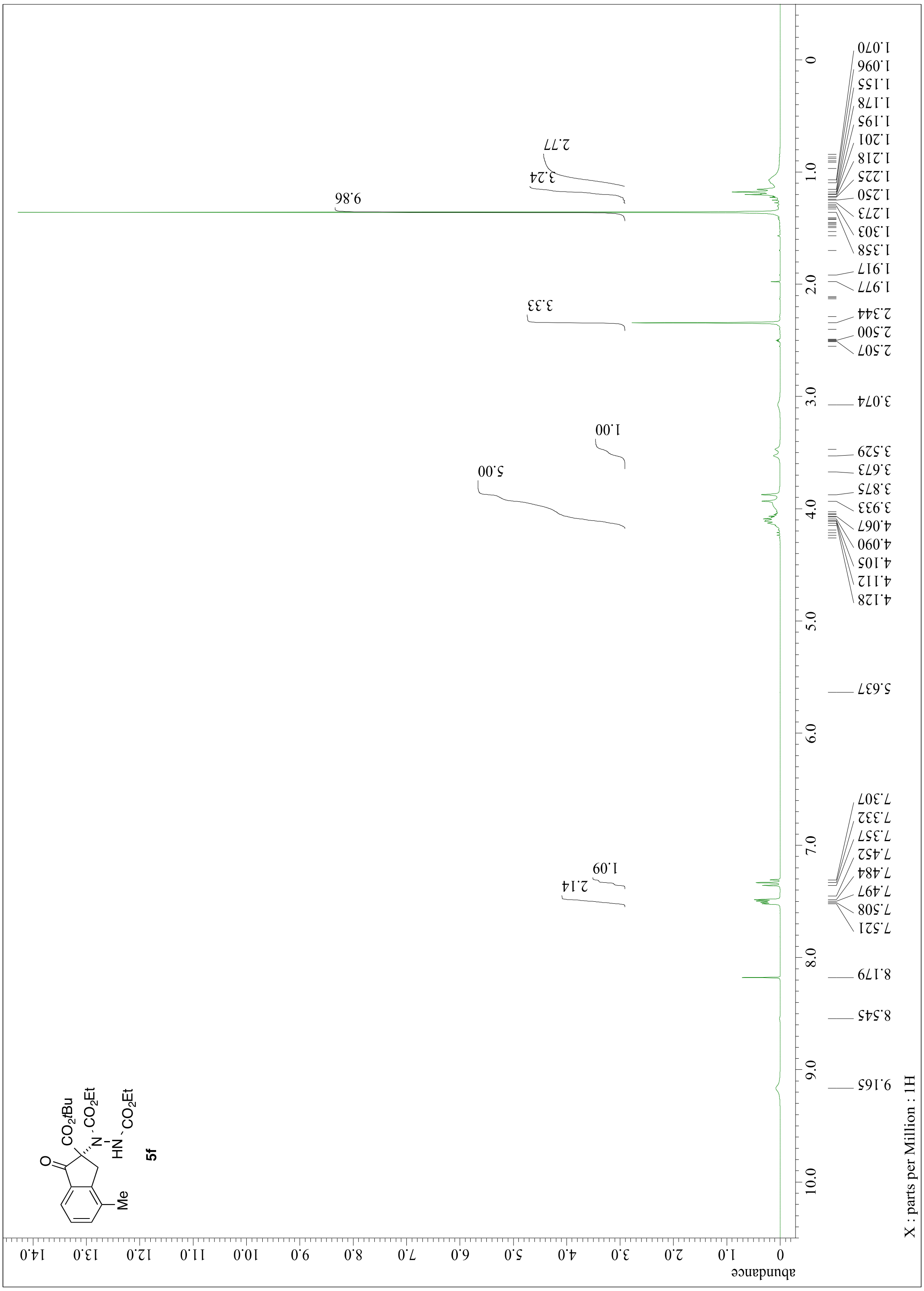

CO2tBu
N CO2Et
HN CO2Et
Me
5f
9.86
5.00
3.33
3.24
2.77
2.14
1.09
1.00
9.165
8.545
8.179
7.521
7.508
7.497
7.484
7.452
7.357
7.332
7.307
5.637
4.128
4.112
4.105
4.090
4.067
3.933
3.875
3.673
3.529
3.074
2.507
2.500
2.344
1.977
1.917
1.358
1.303
1.273
1.250
1.225
1.218
1.201
1.195
1.178
1.155
1.096
1.070
abundance
X : parts per Million : 1H

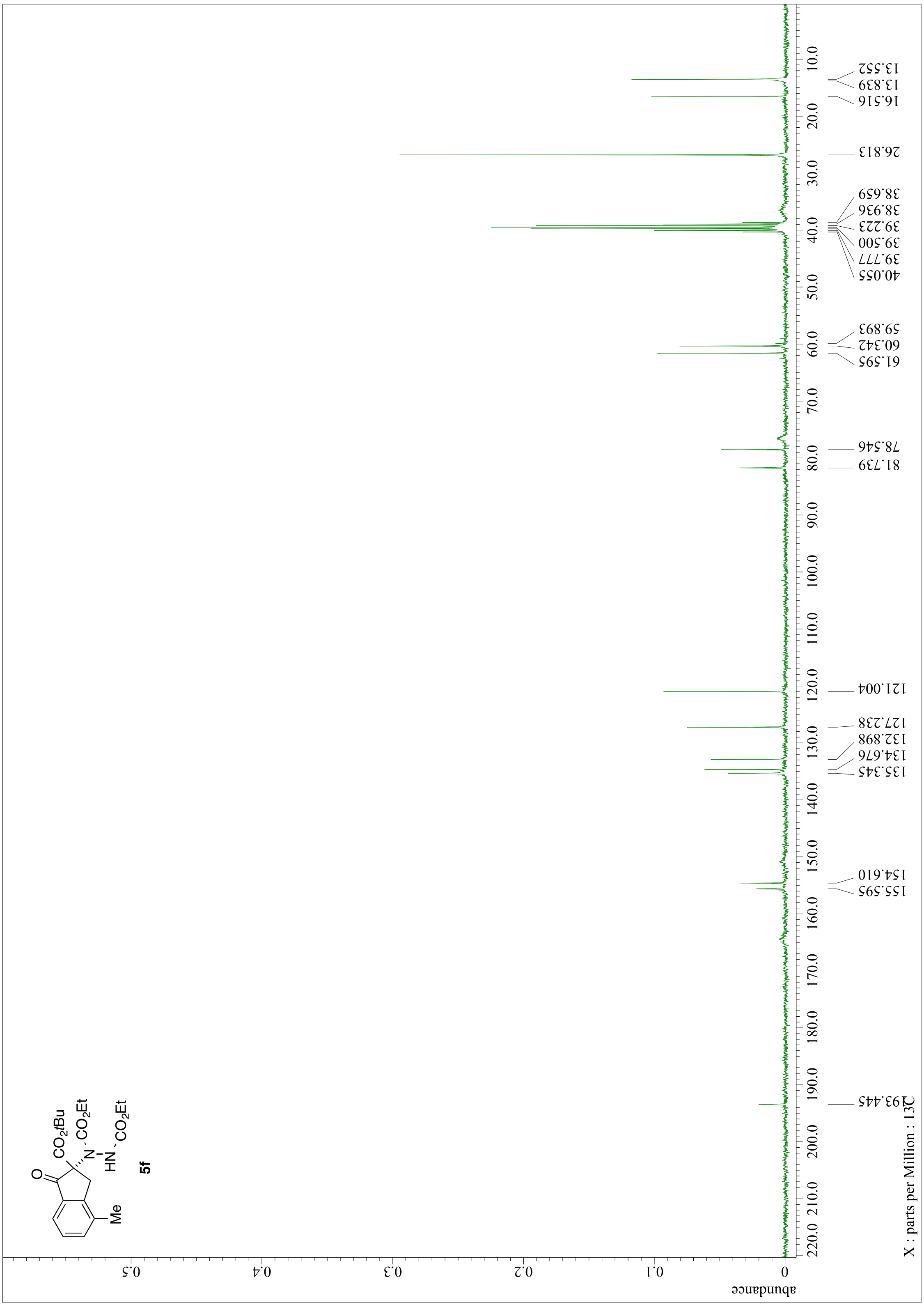

5f
Me
CO2tBu
CO2Et
CO2Et
HN
N
O
13.552
13.839
16.516
26.813
38.659
38.936
39.223
39.500
39.777
40.055
59.893
60.342
61.595
78.546
81.739
121.004
127.238
132.898
134.676
135.345
154.610
155.595
193.445
X : parts per Million : 13C
abundance

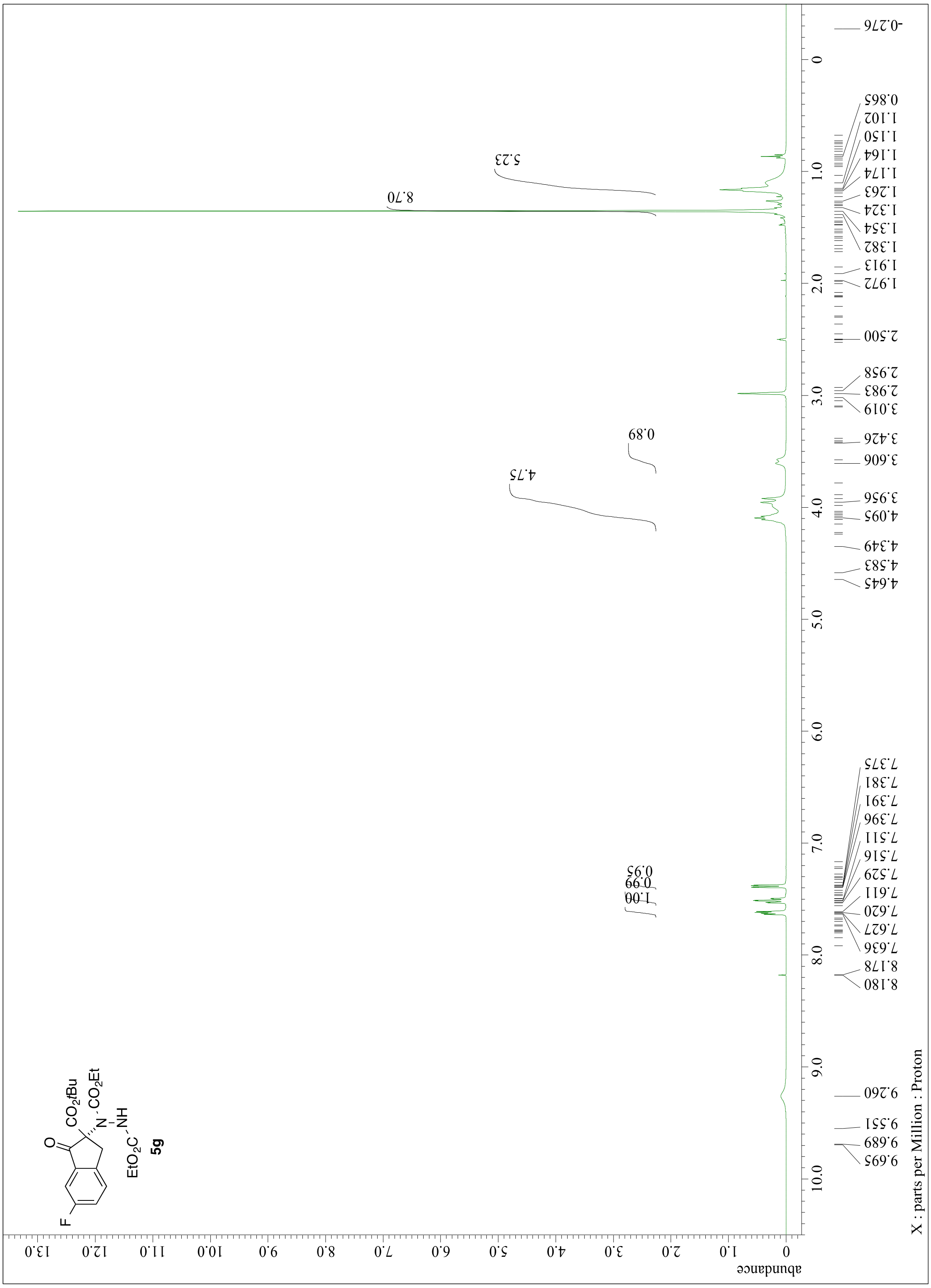

5g
X : parts per Million : Proton
abundance
-0.276
0.865
1.102
1.150
1.164
1.174
1.263
1.324
1.354
1.382
1.913
1.972
2.500
2.958
2.983
3.019
3.426
3.606
3.956
4.095
4.349
4.583
4.645
7.375
7.381
7.391
7.396
7.511
7.516
7.529
7.611
7.620
7.627
7.636
8.178
8.180
9.260
9.551
9.689
9.695
5.23
8.70
0.89
4.75
0.95
0.99
1.00

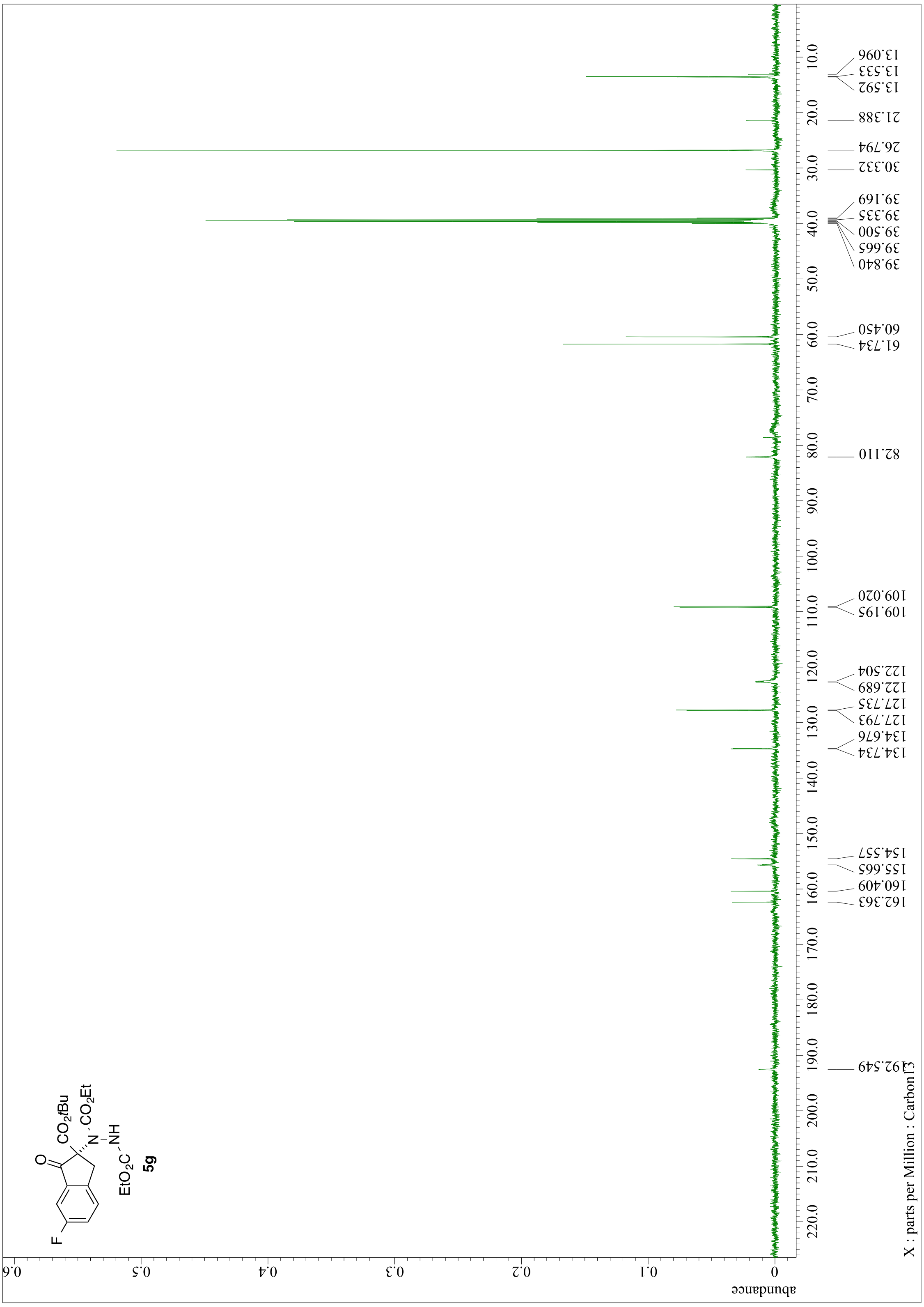

13.096
13.533
13.592
21.388
26.794
30.332
39.169
39.335
39.500
39.665
39.840
60.450
61.734
82.110
109.020
109.195
122.504
122.689
127.735
127.793
134.676
134.734
154.557
155.665
160.409
162.363
192.549
X : parts per Million : Carbon13
abundance
5g

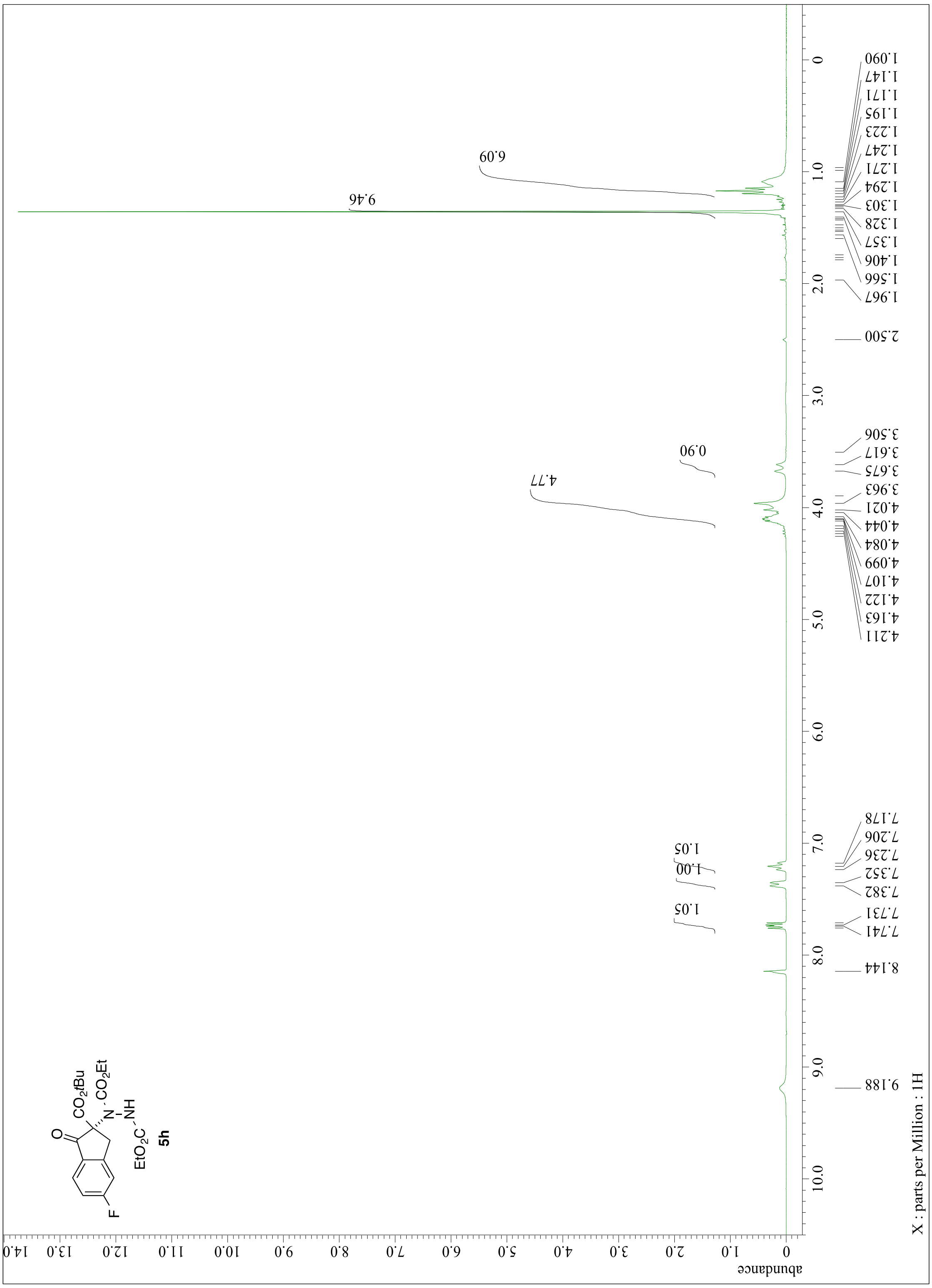
5h
X : parts per Million : 1H
abundance
9.188
8.144
7.741
7.731
7.382
7.352
7.236
7.206
7.178
4.211
4.163
4.122
4.107
4.099
4.084
4.044
4.021
3.963
3.675
3.617
3.506
2.500
1.967
1.566
1.406
1.357
1.328
1.303
1.294
1.271
1.247
1.223
1.195
1.171
1.147
1.090
9.46
6.09
4.77
0.90
1.05
1.00
1.05

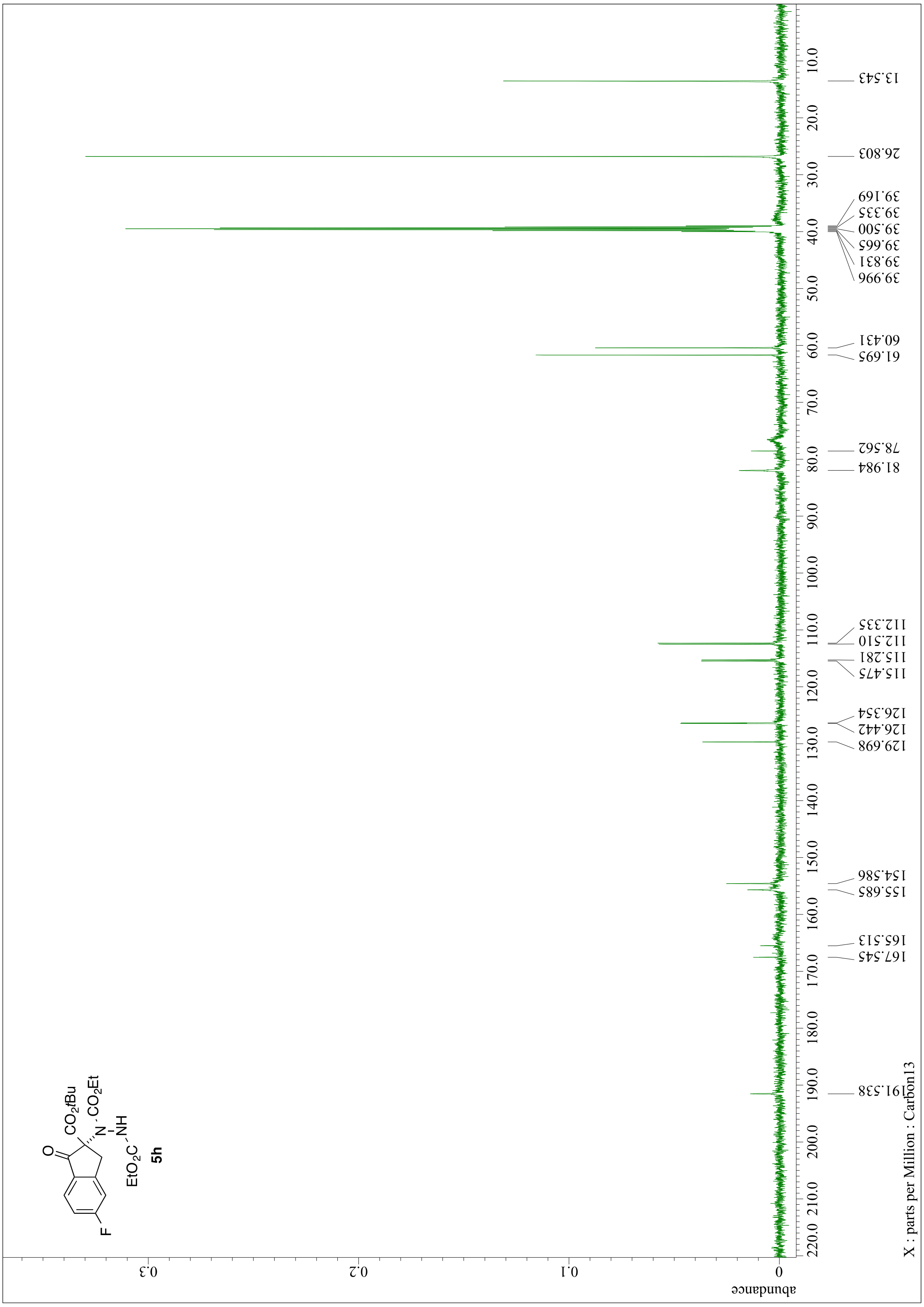
13.543
26.803
39.169
39.335
39.500
39.665
39.831
39.996
60.431
61.695
78.562
81.984
112.335
112.510
115.281
115.475
126.354
126.442
129.698
154.586
155.685
165.513
167.545
191.538
X : parts per Million : Carbon13
abundance
5h

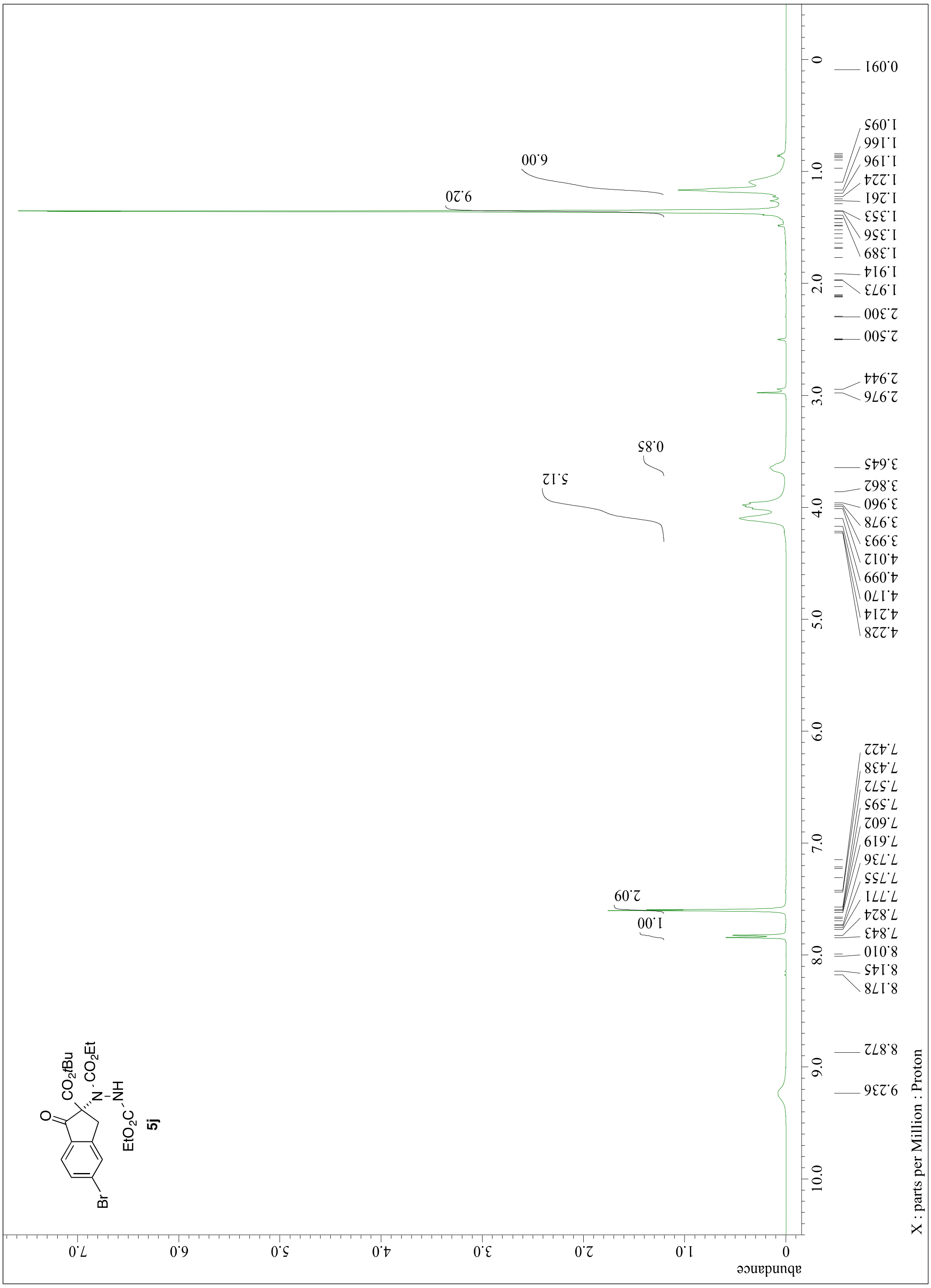
5j
X : parts per Million : Proton
abundance
0.091
1.095
1.166
1.196
1.224
1.261
1.353
1.356
1.389
1.914
1.973
2.300
2.500
2.944
2.976
3.645
3.862
3.960
3.978
3.993
4.012
4.099
4.170
4.214
4.228
7.422
7.438
7.572
7.595
7.602
7.619
7.736
7.755
7.771
7.824
7.843
8.010
8.145
8.178
8.872
9.236
6.00
9.20
0.85
5.12
2.09
1.00

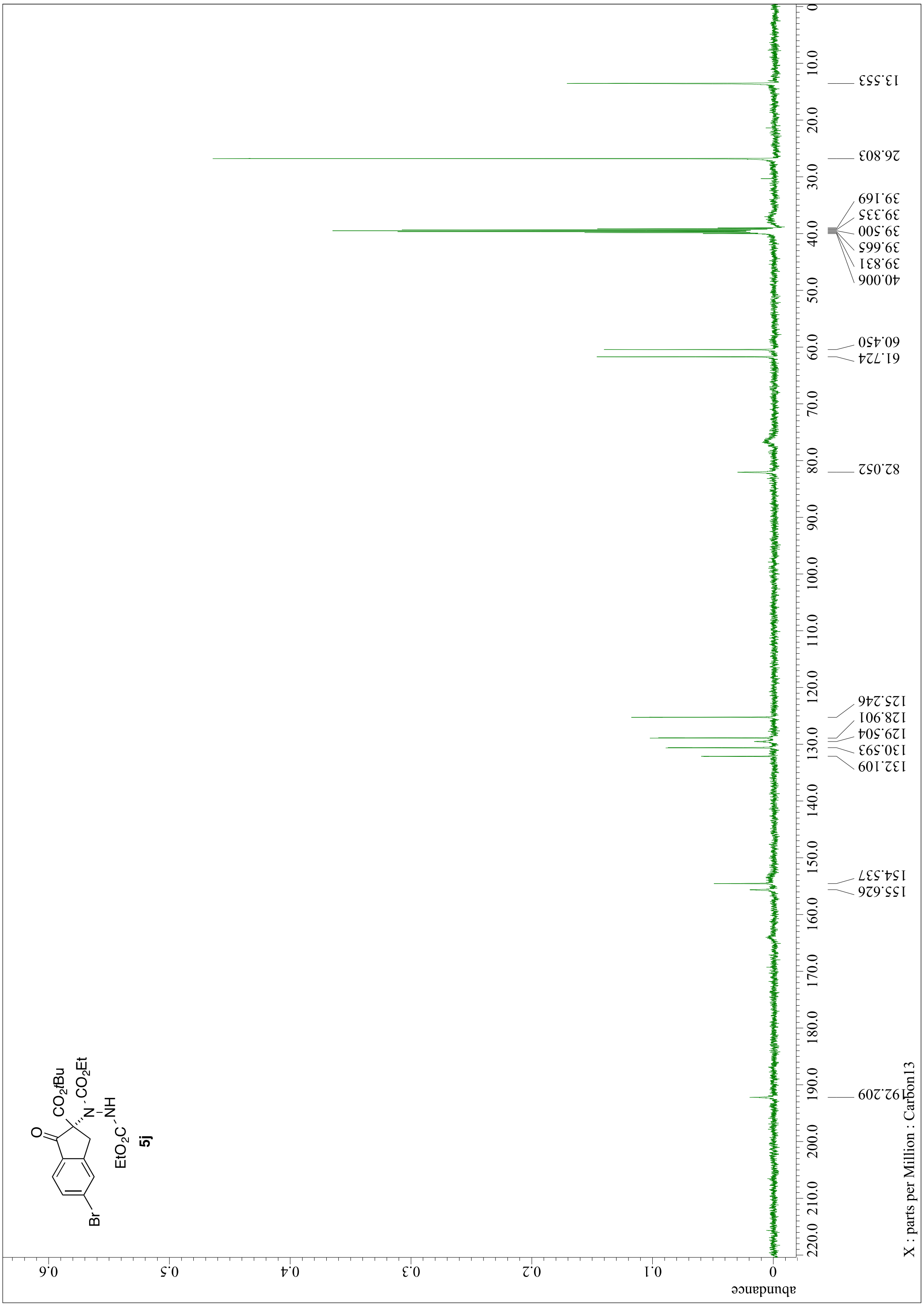
13.553
26.803
39.169
39.335
39.500
39.665
39.831
40.006
60.450
61.724
82.052
125.246
128.901
129.504
130.593
132.109
154.537
155.626
192.209
X : parts per Million : Carbon13
abundance
5j
